# Biotechnological potential of red yeast isolated from birch forests in Poland

**DOI:** 10.1007/s10529-024-03482-3

**Published:** 2024-04-30

**Authors:** Anna M. Kot, Paulina Laszek, Marek Kieliszek, Katarzyna Pobiega, Stanisław Błażejak

**Affiliations:** Department of Food Biotechnology and Microbiology, Institute of Food Sciences, Warsaw University of Life Sciences, Nowoursynowska 159C, 02-776 Warsaw, Poland

**Keywords:** Biocontrol, Carotenoids, Enzymes, Exopolysaccharides, Microbial oil, Red yeast

## Abstract

**Objectives:**

This study aimed to isolate red yeast from sap, bark and slime exudates collected from Polish birch forests and then assessment of their biotechnological potential.

**Results:**

24 strains of red yeast were isolated from the bark, sap and spring slime fluxes of birch (*Betula pendula*). Strains belonging to *Rhodotorula mucilaginosa* (6), *Rhodosporidiobolus colostri* (4), *Cystrofilobasidium capitaum* (3), *Phaffia rhodozyma* (3) and *Cystobasidium psychroaquaticum* (3) were dominant. The highest efficiency of carotenoid biosynthesis (5.04 mg L^−1^) was obtained by *R. mucilaginosa* CMIFS 004, while lipids were most efficiently produced by two strains of *P. rhodozyma* (5.40 and 5.33 g L^−1^). The highest amount of exopolysaccharides (3.75 g L^−1^) was produced by the *R. glutinis* CMIFS 103. Eleven strains showed lipolytic activity, nine amylolytic activity, and only two proteolytic activity. The presence of biosurfactants was not found. The growth of most species of pathogenic moulds was best inhibited by *Rhodotorula* yeasts.

**Conclusion:**

Silver birch is a good natural source for the isolation of new strains of red yeast with wide biotechnological potential.

## Introduction

The term “red yeast” is used to refer to species that produce large amounts of carotenoids, which are responsible for the orange, pink or red colour of their colonies. These microorganisms show great biotechnological potential, and for this reason, in recent years, they have attracted the interest of the food, pharmaceutical, cosmetic, chemical and feed industries (Li et al. 2022b). Their advantage is the ability to biotransform many carbon sources into various primary and secondary metabolites and the fact that they are widely distributed in nature. Among the well-known red yeasts, there are species belonging to genera *Rhodotorula, Rhodosporidium, Phaffia, Cystofilobasidium, Cystobasidium* and *Sporobolomyces* (Mannazzu et al. 2015)*.* The biotechnological potential of red yeast includes the biosynthesis of microbial oils (Osman et al. 2022), carotenoids (Silva et al. 2023), lipases (Taskin et al. 2016), amylases (Kwon et al. 2020), exopolysaccharides (Wang et al. 2020), biosurfactants (Lin et al. 2022), or biocontrol factors (Ianiri et al. 2017).

Such a wide potential of this group of yeasts means that new strains of red yeast in the natural environment are constantly being sought. An interesting source of isolation is the fluxes of deciduous trees. There are two types of exudates: long-lasting (chronic slime-fluxes) and short-lived (spring sap-flows). Chronic exudates are caused by mechanical injuries and the activity of pathogenic invertebrates and bacteria (Weber 2006). Most often, they are caused by bacteria that infect wounds and get under the bark of the tree, where they proliferate and then carry out the fermentation process. The carbon dioxide produced increases the pressure and pushes the juice into the crack, causing it to flow out (fluxing). As a result of the leak coming into contact with air, various microorganisms begin to develop in it, especially yeasts that produce mucous substances (Kerrigan et al. 2004). Spring exudates are formed in places of damage to the bark of the trunk or branches. During the bud burst period, the juices contain the highest amounts of sugars and other nutrients and are rapidly colonised by microorganisms once they have flowed out. Birch trees (*Betula* spp.) are particularly efficient juice producers, and their juice, rich in simple sugars (up to 1% w/v), is increasingly consumed by consumers (Weber 2006).

From the exudates of various trees, yeasts such as *Botryozyma mucatilis* (Kerrigan et al. 2004), *Rhodotorula bacarum, Fibulobasidium inconspicuum, Pichia anomala, Sporidiobolus ruineniae* (Mushtaq et al. 2004), *Bullera pseudoalba*, *Candida lyxosophila*, *Cryptococcus gasrticus*, *Pichia anomala*, *P. strasburgensis*, *Wiliopsis californica* (Mushtaq and Hashmi 2008), *Xanthophyllomyces dendrorhous, Hanseniaspora uvarum, Aureobasidium pullulans* and *Cystofilobasidium infirmominiatum* were isolated (Weber et al. 2006).

These studies prove that the forest environment can be a rich source of interesting microorganisms with biotechnological potential. To the best of the authors’ knowledge, no studies have been conducted on the isolation of red yeast from birch forests in Poland so far. Isolation of microorganisms from weakly studied environments may provide new strains with interesting biotechnological properties and also increase the number of strains available in national collections of microorganisms for other researchers. The aim of the these research was to isolate red yeast from sap, bark and mucous exudates collected from Polish birch forests and then to assess the ability of these yeasts to biosynthesis carotenoids, lipids, exopolysaccharides, enzymes, as well as their antagonistic potential against selected fungal pathogens.

## Materials and methods

### Red yeast isolation

During the spring seasons of 2020 and 2021, a total of 36 samples of sap, bark and slime fluxes from silver birch (*Betula pendula*) were collected from five birch forests located near Skarżysko-Kamienna (51° 06′ 47″ N, 20° 52′ 17″ E), Ryki (51° 37′ 32″ N, 21° 55′ 57″ E), Grudziądz (53° 29′ 02″ N, 18° 45′ 13″ E), Wyszków (52° 35′ 34″ N, 21° 27′ 30″ E) and Otwock (52° 06′ 20″ N, 21° 15′ 40″ E). Samples were immediately transferred to a liquid YPD medium (2% glucose, 2% peptone, 1% yeast extract, pH 5.6), transported to the laboratory and incubated for 3 days at 22 °C. After this time, the culture was transplanted into Sabouraud Chloramphenicol Agar using a loop. The plates were incubated at 22 °C for 4–7 days. Yeasts with orange, red or pink coloured colonies were then reductively subcultured into Sabouraud Chloramphenicol Agar until pure cultures were obtained.

### Red yeast identification

DNA was isolated by the chloroform-phenol method. Pure yeast culture was taken from the agar medium with a loop and suspended in 300 μL of lysis buffer (1 mM EDTA, 10 mM Tris–HCl, 100 mM NaCl, 1% Triton X-100, 1% SDS, pH 8.0) and then incubated at 37 °C. After an hour of incubation, 200 μl of TE buffer (10 mM Tris–HCl, pH 8.0; 1 mM EDTA) was added and mixed vigorously for one minute. Then 200 μL of a mixture of phenol:chloroform:isoamyl alcohol (25:24:1, pH 8.0) was added, mixed and centrifuged (8500×*g* for 10 min). The top layer was collected, and the DNA was precipitated by adding 600 μL of cold 96% ethanol and incubating at − 18 °C for 30 min. The samples were centrifuged for 10 min at 14,000 rpm, and then the pellet was washed with 500 μL of 70% ethanol and centrifuged again at the same parameters. The ethanol was removed, and the pellet was resuspended in 20 μL of sterile, nuclease-free PCR water. The first step of the PCR reaction was to prepare a mixture that contained primers (20 pmol each), dNTP (0.2 mM), MgCl_2_ (1.5 mM), Taq polymerase (1 U). 1 μL of 250–300 ng μL^−1^ DNA solution was added to the mixture. Primers NL1 (5′-GCATATCAATAAGCGGAGGAAAAG-3′) and NL4 (5′-GGTCCGTGTTTCAAGACGG-3′) were used. The PCR process was run with the following parameters: initial denaturation at 94 °C (2 min), 36 cycles: denaturation at 91 °C (1 min), hybridisation at 60 °C (1 min), elongation at 72 °C (1 min), and at the end of the process, a single final extension at 72 °C for 5 min was applied. The PCR samples were sent to the Genomed laboratory (Warsaw) to determine the sequence of the LSU domain. The obtained sequences were analysed in the BLAST program and compared with sequences deposited at the National Center for Biotechnology Information. Based on the obtained results, the sequences of the new red yeast isolates were deposited in GenBank (accession numbers are given in Table 1). The strains were included in the Collection of Microorganisms of the Institute of Food Science (CMIFS) of the Warsaw University of Life Sciences.

**Table 1:** List of red yeast strains isolated from *Betula pendula* with source, place of isolation and GenBank accession numbers

Isolate number	Source	Location	Identification (GenBank acc. number)	Identity (%)	CMIFS collection number
A-001	Birch sap	Grudziądz (53° 29′ 02″ N, 18° 45′ 13″ E)	*Rhodotorula mucilaginosa* (OQ297751)	99.83	097
A-002	Birch sap	Wyszków (52° 35′ 34″ N, 21° 27′ 30″ E)	*Rhodotorula mucilaginosa* (OQ297789)	100.0	098
A-003	Birch bark	Skarżysko-Kamienna (51° 06′ 47″ N, 20° 52′ 17″ E)	*Rhodotorula mucilaginosa* (OM256524)	100.0	004
A-004	Birch bark	Otwock (52° 06′ 20″ N, 21° 15′ 40″ E)	*Rhodotorula mucilaginosa* (OQ299502)	100.0	099
A-005	Birch bark	Skarżysko-Kamienna (51° 06′ 47″ N, 20° 52′ 17″ E)	*Rhodotorula mucilaginosa* (OQ299503)	100.0	100
A-006	Birch bark	Skarżysko-Kamienna (51° 06′ 47″ N, 20° 52′ 17″ E)	*Rhodotorula mucilaginosa* (OQ299505)	100.0	101
A-007	Birch slime flux	Skarżysko-Kamienna (51° 06′ 47″ N, 20° 52′ 17″ E)	*Phaffia rhodozyma* (OR582335)	100.0	102
A-008	Birch slime flux	Skarżysko-Kamienna (51° 06′ 47″ N, 20° 52′ 17″ E)	*Rhodotorula glutinis* (OQ996827)	100.0	103
A-115	Birch slime flux	Skarżysko-Kamienna (51° 06′ 47″ N, 20° 52′ 17″ E)	*Cystofilobasidium capitatum* (OQ996833)	100.0	104
A-116	Birch slime flux	Skarżysko-Kamienna (51° 06′ 47″ N, 20° 52′ 17″ E)	*Buckleyzyma aurantiaca* (OQ297788)	100.0	105
A-117	Birch slime flux	Skarżysko-Kamienna (51° 06′ 47″ N, 20° 52′ 17″ E)	*Rhodosporidiobolus colostri* (OQ299506)	100.0	106
A-118	Birch slime flux	Skarżysko-Kamienna (51° 06′ 47″ N, 20° 52′ 17″ E)	*Cystobasidium psychroaquaticum* (OQ299510)	100.0	107
A-119	Birch slime flux	Skarżysko-Kamienna (51° 06′ 47″ N, 20° 52′ 17″ E)	*Symmetrospora coprosmae* (OQ996834)	100.0	108
A-120	Birch slime flux	Ryki (51° 37′ 32″ N, 21° 55′ 57″ E)	*Rhodotorula babjevae* (OR018113)	99.83	109
A-121	Birch slime flux	Ryki (51° 37′ 32″ N, 21° 55′ 57″ E)	*Phaffia rhodozyma* (OR582722)	100.0	110
A-122	Birch slime flux	Ryki (51° 37′ 32″ N, 21° 55′ 57″ E)	*Cystobasidium psychroaquaticum* (OQ299511)	100.0	111
A-123	Birch slime flux	Ryki (51° 37′ 32″ N, 21° 55′ 57″ E)	*Cystobasidium psychroaquaticum* (OQ299512)	99.83	112
A-124	Birch slime flux	Ryki (51° 37′ 32″ N, 21° 55′ 57″ E)	*Cystofilobasidium capitatum* (OQ999905)	100.0	113
A-125	Birch slime flux	Wyszków (52° 35′ 34″ N, 21° 27′ 30″ E)	*Phaffia rhodozyma* (OR582723)	100.0	114
A-126	Birch slime flux	Wyszków (52° 35′ 34″ N, 21° 27′ 30″ E)	*Rhodosporidiobolus colostri* (OQ299543)	100.0	115
A-127	Birch slime flux	Wyszków (52° 35′ 34″ N, 21° 27′ 30″ E)	*Cystofilobasidium infirmominiatum* (OQ299546)	100.0	116
A-128	Birch slime flux	Wyszków (52° 35′ 34″ N, 21° 27′ 30″ E)	*Rhodosporidiobolus colostri* (OQ299545)	99.83	117
A-129	Birch slime flux	Grudziądz (53° 29′ 02″ N, 18° 45′ 13″ E)	*Rhodosporidiobolus colostri* (OQ299547)	100.0	118
A-130	Birch slime flux	Grudziądz (53° 29′ 02″ N, 18° 45′ 13″ E)	*Cystofilobasidium capitatum* (OR018114)	100.0	119

### Carotenoid biosynthesis

The yeast was inoculated into a liquid medium composed of: 40 g glucose L^−1^, 20 g peptone L^−1^, 10 g yeast extract L^−1^, pH 5.6. Cultures were grown on a shaker (140 rpm, 22 °C) for 96 h. After this time, the biomass was centrifuged, 0.5 g of glass beads with a diameter of 0.5 mm and 2 mL of DMSO were added to it and then stirred (70 rpm) for 1 h. After this time, 2 mL of petroleum ether, 2 mL of acetone, and 2 mL of 20% sodium chloride were added and stirred again for an hour. To separate the phases, the mixture was centrifuged (2600×*g* for 5 min), the ether phase (containing carotenoids) was removed, and its absorbance was measured at 490 nm against petroleum ether. The results expressed as total carotenoid content in dry cell biomass (μg/g) were calculated by the formula = A_max_ *x* D *x* V/E *x* W, where: A_max_—absorbance at 490 nm; D—sample dilution factor; V—volume of the ether phase; E—carotenoid extinction coefficient (0.16); W—grams of dry cellular substance (g) (Cheng and Yang 2016).

The carotenoid profile was analysed by high-performance liquid chromatography. The extraction solvent was evaporated under nitrogen, and carotenoids were suspended in the HPLC phase and filtered through a 0.45 μm filter. Separation was carried out on an analytical column C18 Luna HILIC—Phenomenex (250 mm × 4.6 mm, 5 μm). Elution conditions were: flow 0.7 mL min^−1^, temperature 25 °C, wavelength 490 nm. The mobile phase consisted of acetonitrile, isopropanol and ethyl acetate in a ratio of 4:4:2 (v/v). HPLC analysis was carried out in isocratic flow (Bhosale and Gadre 2001). For the strains identified as *P. rhodozyma*, a different composition of the mobile phase was used: acetonitrile and ultrapure water in a ratio of 95:5 (v/v), isocratic flow of 0.8 mL min^−1^ and a lower wavelength: 474 nm (Kanwugu et al. 2020). After the analysis was completed, the areas of the respective peaks were determined and based on them, the percentages of individual carotenoid fractions were determined.

### Biosynthesis of exopolysaccharides

Yeast was inoculated into a liquid medium with the following composition: 50 g sucrose L^−1^, 2 g (NH_4_)_2_SO_4_ L^−1^, 1 g KH_2_PO_4_ L^−1^, 0.5 g MgSO_4_·7H_2_O L^−1^, 0.1 g CaCl_2_·2H_2_O L^−1^, 0.1 g NaCl L^−1^, 1 g yeast extract L^−1^, pH 5.6. Cultivation was carried out on a shaker (140 rpm) at 22 °C for 96 h. The yeast culture medium was centrifuged at 8228×*g* for 30 min. Then, 20 mL of 96% ethanol was added to 10 mL of the supernatant and left for 24 h at 4 °C to precipitate the polymers. After this time, the precipitated polymers were centrifuged (8228×*g* for 10 min), the supernatant was removed, and the resulting precipitate was dried at 80 °C to a constant weight. The dried sludge was weighed on an analytical balance, and the results were given in grams per litre of medium (Gientka et al. 2016).

### Lipid biosynthesis

A liquid medium was prepared with the following composition: 40 g glucose L^−1^, 1 g MgSO_4_·7 H_2_O L^−1^, 1,5 g KH_2_PO_4_ L^−1^, 0,15 g CaCl_2_ L^−1^, 0,1 g FeSO_4_·7 H_2_O L^−1^, 1 g Na_2_HPO_4_·12 H_2_O L^−1^, 0,1 g CuSO_4_·5 H_2_O L^−1^, 0,1 g MnSO_4_·H_2_O L^−1^, 1,0 g yeast extract L^−1^, 0,5 g peptone L^−1^ and 0,11 g (NH_4_)_2_HPO_4_ L^−1^, pH 5,6. The initial C/N molar ratio was 70. For yeast cultivation, the flasks were placed in a shaker (140 rpm) and grown at 22 °C for 96 h. After this time, the yeast biomass was separated from the substrate by centrifugation (8228×*g* for 10 min), dried (85 °C/24 h) and ground. Lipid content was determined by Bligh and Dyer’s gravimetric extraction method with modifications. In order to disintegrate the cell wall, 200 mg of dry biomass was weighed, and 10 mL of 1 M HCl was added to it. The samples were incubated for 2 h at 60 °C. After cooling, 5 mL of chloroform and 10 mL of methanol were added to the samples and shaken vigorously for 30 min. Then, 5 mL of chloroform and 5 mL of 20% NaCl were added and shaken again for 30 min. The entire mixture was centrifuged (3214×*g* for 10 min), and the lower chloroform layer containing the lipids was collected and transferred to a new tube. The chloroform was evaporated under nitrogen, and the total lipid content was reported in g 100 g^−1^ (Zhang et al. 2011; Kot et al. 2020). The extracted lipids were suspended in hexane, and a solution of 2M KOH in methanol was added (incubation 37°C/night). After this time, the clear hexane layer was transferred to a chromatographic roller and analysed in a gas chromatograph (GC-FID, TRACE™ 1300, Thermo Scientific, USA) using an RTX-2330 capillary column (60 m × 0.25 mm × 0.2 μm, Restek, USA). The chromatograph oven temperature was set at 50 °C (3 min), followed by a temperature increase of 3 °C/min to 250 °C (5 min). The carrier gas was nitrogen (1.6 mL min^−1^). The detector was operated at 260 °C (Kot et al. 2019a). Fatty acid identification was based on the retention time of standards from Nu-Chek-Prep Inc. (USA), and the percentage of fatty acids in the lipid fraction was calculated.

### Lipolytic, cellulolytic, amylolytic and proteolytic activity

Media were prepared to determine the ability to produce lipolytic enzymes (5 g tributyrin L^−1^, 5 g peptone L^−1^, 3 g yeast extract L^−1^, 20 g agar L^−1^, pH 7.0), cellulolytic (10 g carboxymethylcellulose L^−1^, 10 g Na_2_CO_3_ L^−1^, 5 g peptone L^−1^, 5 g yeast extract L^−1^, 5 g NaCl L^−1^, 1 g KH_2_PO_4_ L^−1^, 2 g MgSO_4_·7H_2_O L^−1^, 20 g agar L^−1^, pH 9.5), amylolytic (10 g peptone L^−1^, 2 g starch L^−1^, 20 g agar L^−1^, pH 5.6) and proteolytic (10 g peptone L^−1^, 2 g yeast extract L^−1^, 40 g gelatin L^−1^, 20 g agar L^−1^, pH 7.6). After sterilisation, the medium was poured into Petri dishes, and after solidification, sterile discs with a diameter of 6 mm soaked in yeast suspension (1 × 10^6^ CFU mL^−1^) were placed and incubated for 7 days at 22 °C. In the case of the medium with tributyrin, lipolytic activity was indicated by the appearance of clear zones around the colonies. Cellulolytic activity was indicated by clear zones around the colonies after flooding with Congo red solution, while amylolytic activity was indicated by the appearance of yellow/orange zones after flooding with Lugol's solution. Proteolytic degradation was detected using a sublimate (mercuric (II) chloride solution), which reacted with undegraded protein, giving a milky-coloured precipitate. Zones of clear medium formed around colonies with proteolytic properties. The results were expressed as: − negative result, no enzymatic properties; + positive result, the diameter indicating a positive result of a given method was less than 1 cm (after subtracting the diameter of the paper disc); ++ positive result, the diameter indicating a positive result of a given method was smaller than 1 cm and smaller than 2 cm (after subtracting the diameter of the paper disc); +++ positive result, the diameter indicating a positive result of a given method was less than 2 cm (after subtracting the diameter of the paper disc).

### Biosynthesis of biosurfactants

Yeast was inoculated with a loop on a liquid medium (0.5 g MgSO_4_ L^−1^, 1 g KH_2_PO_4_ L^−1^, 0.01 g FeSO_4_ L^−1^, 1.5 g (NH_4_)_2_SO_4_ L^−1^, 0.02 g CaCl_2_ L^−1^, 1 g peptone L^−1^, 1 g yeast extract L^−1^ and 80 g soybean oil L^−1^, pH 5.6). The flasks were placed in a shaker (140 rpm) at 22 °C for 96 h. After the cultivation, the yeast biomass was centrifuged for 30 min at 8228 × g, and the supernatant was left for determination. Three tests were performed to determine the presence of biosurfactants (Eldin et al. 2019; Patel and Patel 2020):

- Parafilm-M test: 25 μL of the supernatant was dotted onto Parafilm, and the shape of the droplets was evaluated after one minute. If it was flat, did not retain its shape, it indicated the presence of biosurfactants.
- Oil displacement test: 10 mL of soybean oil was placed on the surface of 30 mL of distilled water, and then 1 mL of supernatant was applied to the centre of the oil film. A negative test was performed in the same way − 1 mL of water was added instead of the supernatant. Within 30 s, oil displacement and the formation of a clean, clear zone around the supernatant containing biosurfactants were observed. After the test, the diameter of this zone was measured, and the surface area in cm^2^ was calculated.
- Phenol-sulphur test. For this purpose, 1 mL of phenol solution (5% w/v) was added to 1 mL of the supernatant, and then 2–5 mL of concentrated H_2_SO_4_ acid was added dropwise until the characteristic orange colour appeared. The mixture was shaken and left for 10 min at room temperature. The colour change from yellow to orange indicated the presence of glycolipid biosurfactants.

### The antifungal activity

The study of the antimicrobial activity of all tested red yeast strains was carried out against five test mould strains: *Aspergillus niger* ATCC 9142, *Fusarium solani* ATCC 36031, *Botritis cinerea* IOR 2193, *Penicillium expansum* CCM F-576 and *Alternaria solani* DSM 2947. Strains marked with ATCC symbols were purchased from the American Type Culture Collection (USA), IOR from Instytut Ochrony Środowiska in Poznań (Poland), CCM from the Czech Collection of Microorganisms (Czech Republic), and DSM from the German Collection of Microorganisms and Cell Cultures (Germany). The YPD agar medium was surface inoculated with red yeast suspension (1 × 10^6^ CFU mL^−1^). Then, a cut disc of the medium with abundant mould growth (6 mm in diameter) was transferred to the centre. At the same time, control plates were prepared—discs with mycelium were transferred to Petri dishes without yeast. All plates were incubated at 22 °C for 7 days. After this time, the mould growth diameter was measured and compared with the control sample (Shen et al. 2019).

### Statistical analysis

The mean and standard deviation were calculated for the obtained results, and statistical analysis was performed in the R program (version 8.9). For this purpose, one-way analysis of variance (ANOVA) and Tukey’s test at the significance level of 0.05 were used.

## Results and discussion

### Yeast identification

**Fig. 1:**
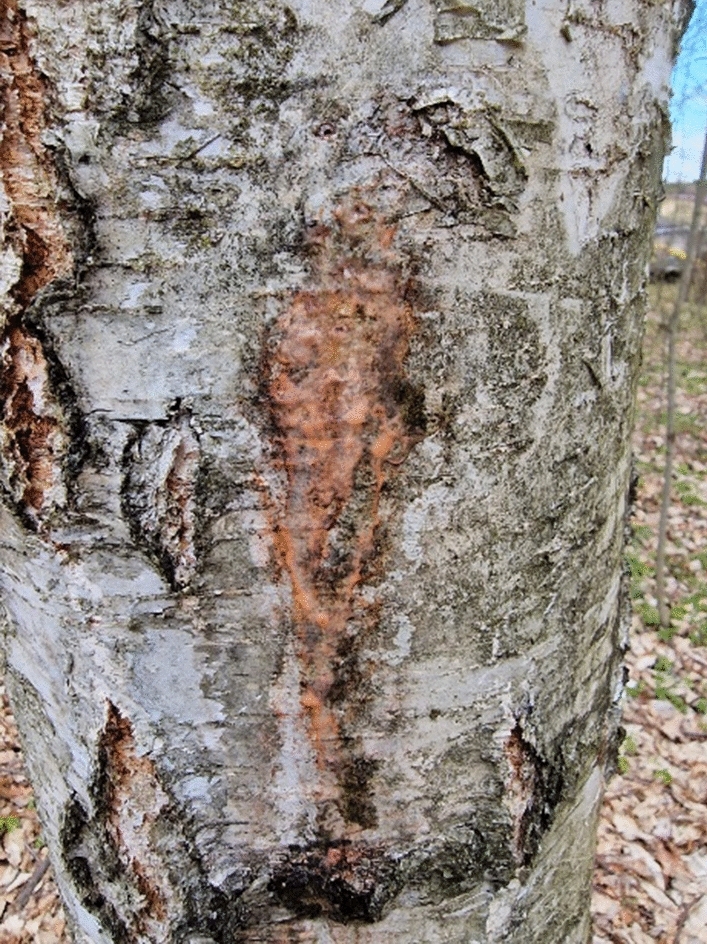
An example of spring birch slime flux. The photo was taken in April 2021 in a forest located near Skarżysko-Kamienna in Poland

As a result of the work, 24 strains of red yeast were isolated. Samples were taken in the spring of 2020 and 2021. Eight strains isolated in 2020 were included in the list of 114 isolates of red yeast, which were obtained as part of research to determine the diversity of these microorganisms in various regions and environments of Poland. One of these eight strains (CMIFS 004) has also been identified as *R. mucilaginosa* and deposited with GenBank under accession number OM256524 (Kot et al. 2023). The remaining 7 strains isolated in 2020 and 16 strains from 2021 were identified in this work (Fig. 1).

Most isolated strains belonged to the genus *Rhodotorula*: *R. mucilaginosa* (6), *R. glutinis* (1) and *R. babjevae* (1) (Table 1). Interestingly, the yeast *R. mucilaginosa* was isolated only from freshly collected birch sap and bark, and was not found in exudates. Colonies of the yeast *R. mucilaginosa* (CMIFS 004, and 097-101) were red or red–orange, smooth and shiny, with small, round or ellipsoid cells that propagated by budding. The yeast *R. glutinis* CMIFS 103 colonies were also red and shiny, and the cells were small and round (Table 2). *R. babjevae* strain CMIFS 109 grew as orange and shiny colonies on the agar medium. *Rhodotorula* yeasts are cosmopolitan microorganisms isolated from various environments, for example, plant leaves, flowers, fruits, soil, mucous secretions from deciduous trees, refinery wastewater and air (Aksu and Eren 2007; El-Banna et al. 2012; de Garcia et al. 2012).

**Table 2:** Morphology of red yeast after 14 days of cultivation on YPD agar medium

Yeast strain (CMIFS number)	Yeast morphology on YPD agar	Yeast strain (CMIFS number)	Yeast morphology on YPD agar
*Rhodotorula mucilaginosa* CMIFS 097		*Rhodotorula mucilaginosa* CMIFS 098	
*Rhodotorula mucilaginosa* CMIFS 004		*Rhodotorula mucilaginosa* CMIFS 099	
*Rhodotorula mucilaginosa* CMIFS 100		*Rhodotorula mucilaginosa* CMIFS 101	
*Phaffia rhodozyma* CMIFS 102		*Rhodotorula glutinis* CMIFS 103	
*Cystofilobasidium capitatum* CMIFS 104		*Buckleyzyma aurantiaca* CMIFS 105	
*Rhodosporidiobolus colostri* CMIFS 106		*Cystobasidium psychroaquaticum* CMIFS 107	
*Symmetrospora coprosmae* CMIFS 108		*Rhodotorula babjevae* CMIFS 109	
*Phaffia rhodozyma* CMIFS 110		*Cystobasidium psychroaquaticum* CMIFS 111	
*Cystobasidium psychroaquaticum* CMIFS 112		*Cystofilobasidium capitatum* CMIFS 113	
*Phaffia rhodozyma* CMIFS 114		*Rhodosporidiobolus colostri* CMIFS 115	
*Cystofilobasidium infirmominiatum* CMIFS 116		*Rhodosporidiobolus colostri* CMIFS 117	
*Rhodosporidiobolus colostri* CMIFS 118		*Cystofilobasidium capitatum* CMIFS 119	

For this purpose 20 uL of yeast suspension (approximately 1 × 10^6^ CFU/mL) was placed in the center of agar plate. After 2 weeks of incubation at 20 °C, the plates were photographed with a digital camera. Microscopic photos were taken using a Nexcope NE610 microscope equipped with a DLT-Cam Pro 1080 camera (1 bar = 20 μm)

The microflora of slime fluxes was much more diverse. In addition to *R. glutinis* CMIFS 103 and *R. babjevae* CMIFS 109, 4 strains belonging to the genus *Cystofilobasidium* have also been identified: three *C. capitatum* (CMIFS 104, 113, 119) and one *C. infirmominiatum* (CMIFS 116). *C. capitatum* colonies were orange, semi-matte or glossy with a smooth surface. Some strains of this genus form hyphae at the periphery of colonies (Sampaio 2011a), as was observed with strain CMIFS 119. A fourth strain (CMIFS 116) belongs to the species *C. infirmominiatum* and formed orange and shiny colonies on the agar medium. Budding oval cells were visible under the microscope. *Cystofilobasidium capitatum* was isolated from the fruiting body of the ascomycetous plant parasite *Cyttaria hariotii*, leaf of clover (*Trifolium* sp.) (Sampaio 2011a) and *Carpinus betulus* L. fluxes in Italy (Weber 2006). *C. infirmominiatum* was also previously isolated from spring tree spills (Golubev et al. 2003; Weber 2006).

Four strains have been included in the genus *Rhodosporididiobolus*. All of them belong to the species *Rhodosporidiobolus colostri*. The colonies of strains CMIFS 106 and 117 were fine and orange, while the other two strains (CMIFS 115 and 118) formed pink-red colonies. The cells of all strains were elongated in shape (Table 2). This species was previously included in *Rhodotorula colostri* and is commonly isolated from plants and decaying organic matter (Sampaio 2011b).

**Fig. 2:**
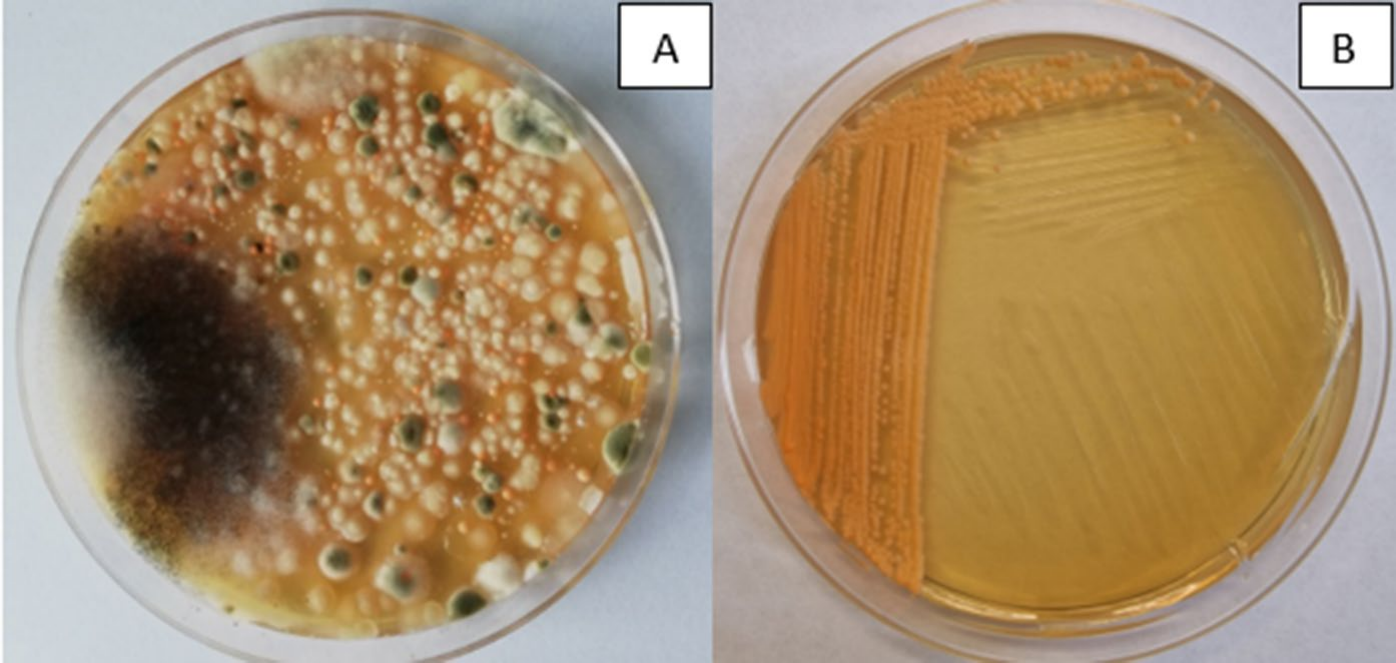
**A** A microbial consortium isolated from spring birch slime flux. The photo shows small orange colonies later identified as *Phaffia rhodozyma* CMIFS 110. **B** Pure *P. rhodozyma* CMIFS 110 yeast colonies after 10 days of cultivation on YPD agar medium at a temperature of 20 °C

Three strains of *Phaffia rhodozyma* have also been identified (Fig. 2). The colonies of these strains were orange. The cells were ellipsoidal, occurring singly, in pairs, and sometimes in chains. *P. rhodozyma* yeast was first isolated from sap leaking from the trunks of deciduous trees growing in the mountainous regions of Alaska and Japan (on the islands of Hokkaido and Honshu) during the 1960s and 1970s (An et al. 1999; Golubev et al. 2003). New strains of this species have also been isolated in other regions, including birch sap spills in European Russia (Libkind et al. 2007) and freshly cut birch spills in Germany (Weber et al. 2006). It was also noted that in many cases in birch exudates from which *P. rhodozyma* yeast was isolated, *Cystofilobasidium* yeasts were also present, e.g. *C. capitatum* and *C. macerans*. Both species belong to the same monophyletic taxon, Cystofilobasidiales (Libkind et al. 2011).

Sequence analysis of CMIFS 107, 111, and 112 strains showed that they belong to the species *Cystobasidium psychroaquaticum*. Their colonies were shiny orange or orange-red. The cells were ovoid, occurring singly or in pairs. This species was introduced into the yeast classification in 2015 by Yurkov and colleagues for a strain originally isolated from leaves of *Chamaedaphne calyculata* in Russia. Most isolates of this species are psychrophilic strains (Yurkov et al. 2015).

In 2015, new types of yeast were separated (Wang et al. 2015), including: *Buckleyzyma, Rhodosporidiobolus* and *Symmetrospora*. Yeasts belonging to these genera have also been isolated from birch exudates. Strain CMIFS 105 was identified as *Buckleyzyma aurantiaca* (syn. *Rhodotorula aurantiaca*). On the agar medium, it grew in the form orange colonies, and the budding cells were ellipsoid in shape. Budding cells were also observed. The yeast *B. aurantiaca* is a species rarely found in the environment, and its occurrence is associated mainly with plants. Strains of this species were isolated from tree leaves and air (Sampaio 2011b).

One strain of the species *Symmetrospora coprosmae* (CMIFS 108) was identified. The colonies of this strain were shiny and intensely pink in colour, while the cells were ellipsoid and propagated by budding. This species was previously classified as *Sporobolomyces coprosmae* (Hamamoto and Nakase 2000) and isolated, e.g. from the leaf surface of *Lactuca sativa* and *Coprosma tenuifolia* (Haelewaters et al. 2020).

### Carotenoid biosynthesis

All tested yeast strains showed the ability to biosynthesise carotenoids (Table 3), and their content was the highest (310.99 μg g^−1^) in the biomass of the *R. mucilaginosa* CMIFS 004 strain. Other isolates belonging to the species *R. mucilaginosa* produced a varied amount of carotenoids ranging from 182.02 (CMIFS 101) to 273.22 μg g^−1^ (CMIFS 100). The *R. glutinis* strain CMIFS 103 produced more than 240 μg g^−1^ of carotenoids, while the *R. babjevae* CMIFS 109 strain only 186.3 μg g^−1^. One of the strains of *R. mucilaginosa* (CMIFS 004) was characterised by the highest volumetric yield − 5.04 mg L^−1^. Other yeasts belonging to the genus *Rhodotorula* have shown yields ranging from 2.48 (CMIFS 109) to 4.20 mg L^−1^ (CMIFS 100). The yield of carotenoid production by red yeast depends on many factors, such as the composition of the medium (e.g. the type of carbon and nitrogen source), the degree of aeration, irradiation or culture temperature. For example, Aksu and Eren (2007) cultivated *R. glutinis* yeast isolated from refinery wastewater using sugar cane molasses as a carbon source. The total content of carotenoids produced increased significantly with increasing aeration rate. With a degree of 1.2 vvm, the volumetric yield of carotenoids was 4.2 mg L^−1^, and with an aeration of 2.4 vvm, 7.1 mg L^−1^ was obtained.

**Table 3:** Carotenoid content, volumetric yield and percentages of carotenoids fraction after 96 h of cultivation of red yeast. Indexes a,b,c… means homogeneous groups determined by Tukey's test (one-way analysis of variance)

Yeast strain (CMIFS number)	Carotenoid content in biomass (µg/g)	Volumetric yield (mg/L)	β-Carotene (%)	Torulene (%)	Torularhodin (%)	γ-Carotene (%)	Astaxanthin (%)
*R. mucilaginosa* 097	199.66 ± 16.1^cdef^	3.15 ± 0.14^cde^	6.87 ± 0.3^hi^	39.61 ± 1.17^bc^	51.78 ± 0.86^fg^	1.75 ± 0.01^ef^	–
*R. mucilaginosa* 098	188.16 ± 6.92^defgh^	3.29 ± 0.06^cde^	6.00 ± 0.22^hij^	41.94 ± 0.77^bc^	50.67 ± 0.95^fg^	1.40 ± 0.04^f^	–
*R. mucilaginosa* 004	310.99 ± 3.24^a^	5.04 ± 0.06^a^	9.72 ± 0.15^gh^	37.86 ± 1.70^c^	50.98 ± 1.77^fg^	1.45 ± 0.22^f^	–
*R. mucilaginosa* 099	215.34 ± 8.45^cdef^	3.50 ± 0.15^c^	3.75 ± 0.53^ijk^	25.76 ± 3.74^d^	69.08 ± 4.12^cde^	1.43 ± 0.15^f^	–
*R. mucilaginosa* 100	273.22 ± 7.59^ab^	4.20 ± 0.08^b^	6.91 ± 0.18^hi^	25.19 ± 0.38^d^	66.89 ± 0.69^de^	1.02 ± 0.12^f^	–
*R. mucilaginosa* 101	182.02 ± 5.29^efghi^	3.00 ± 0.15^ef^	12.43 ± 0.95^fg^	46.96 ± 2.35^b^	39.13 ± 1.14^h^	1.49 ± 0.27^f^	–
*P. rhodozyma* 102	131.08 ± 11.36^jkl^	0.55 ± 0.04^l^	44.80 ± 0.82^a^	7.69 ± 2.32^ijk^	–	8.74 ± 0.06^b^	25.32 ± 0.41^b^
*R. glutinis* 103	240.60 ± 6.11^bc^	3.95 ± 0.1^b^	2.84 ± 0.04^jk^	1.11 ± 0.14^k^	86.82 ± 1.51^a^	9.24 ± 1.32^b^	–
*C. capitatum* 104	147.43 ± 0.97^hijk^	1.51 ± 0.09^k^	16.70 ± 1.64^de^	40.07 ± 1.52^bc^	4.46 ± 1.21^j^	1.89 ± 0.25^ef^	–
*B. aurantiaca* 105	170.77 ± 5.8^fghij^	2.23 ± 0.04^ghi^	14.95 ± 0.92^de^	37.57 ± 0.82^c^	45.19 ± 0.05^gh^	2.30 ± 0.15^def^	–
*R. colostri* 106	190.75 ± 10.94^defgh^	2.59 ± 0.06^ fg^	2.69 ± 0.05^jk^	23.27 ± 1.97^def^	61.97 ± 3.61^e^	2.33 ± 0.66^def^	–
*C. psychroaquaticum* 107	302.97 ± 29.22^a^	1.63 ± 0.07^jk^	3.51 ± 0.7^ijk^	20.77 ± 1.48^defg^	8.87 ± 0.88^j^	4.10 ± 0.66^cde^	–
*S. coprosmae* 108	110.41 ± 4.32^klm^	1.87 ± 0.14^ijk^	2.52 ± 0.18^jk^	14.45 ± 2.58^ghi^	81.54 ± 2.11^ab^	1.50 ± 0.65^f^	–
*R. babjevae* 109	186.3 ± 17.03^defghi^	2.48 ± 0.16^g^	15.62 ± 0.87^ef^	6.12 ± 1.27^jk^	76.29 ± 2.65^bcd^	1.98 ± 0.51^def^	–
*P. rhodozyma* 110	85.63 ± 8.33^m^	0.33 ± 0.02^l^	40.1 ± 0.77^b^	10.89 ± 0.91^hij^	–	4.50 ± 0.21^cd^	35.29 ± 1.49^a^
*C. psychroaquaticum* 111	142.3 ± 11.06^ijk^	1.94 ± 0.07^hij^	2.33 ± 0.44^jk^	17.13 ± 1.53^efgh^	77.48 ± 0.33^abc^	3.07 ± 1.42^cdef^	–
*C. psychroaquaticum* 112	149.92 ± 2.38^hijk^	2.30 ± 0.1^gh^	31.53 ± 1.68^c^	59.25 ± 1.14^a^	6.12 ± 1.18^j^	3.11 ± 0.91^cdef^	–
*C. capitatum* 113	141.14 ± 21.12^ijk^	1.79 ± 0.07^jk^	18.35 ± 0.69^de^	55.44 ± 1.08^a^	9.76 ± 0.16^j^	0.97 ± 0.13^f^	–
*P. rhodozyma* 114	95.24 ± 11.16^lm^	0.52 ± 0.05^l^	46.27 ± 0.65^a^	5.48 ± 0.49^jk^	–	5.01 ± 0.21^c^	35.26 ± 1.44^a^
*R. colostri* 115	151.85 ± 3.58^ghijk^	2.48 ± 0.11^g^	5.86 ± 1.38^hij^	16.31 ± 1.49^fgh^	76.50 ± 0.21^bcd^	1.34 ± 0.33^f^	–
*C. infirmominiatum* 116	185.7 ± 14.41^defghi^	1.73 ± 0.03^jk^	20.13 ± 0.97^d^	11.08 ± 0.95^hij^	23.05 ± 3.49^i^	45.75 ± 1.58^a^	–
*R. colostri* 117	218.77 ± 1.99^cd^	3.07 ± 0.18^de^	1.38 ± 0.23^k^	15.19 ± 3.59^ghi^	79.99 ± 3.53^ab^	3.45 ± 0.29^cdef^	–
*R. colostri* 118	228.82 ± 4.92^bc^	3.48 ± 0.18^cd^	11.72 ± 2.42^fg^	26.02 ± 4.72^d^	60.30 ± 6.98^ef^	1.97 ± 0.16^def^	–
*C. capitatum* 119	195.96 ± 1.01^cdefg^	2.95 ± 0.01^ef^	4.53 ± 1.39^ijk^	24.69 ± 0.41^de^	66.56 ± 1.26^de^	1.01 ± 0.57^f^	–

– not detected

Among the yeasts of the genus *Cystofilobasidium*, the highest amount of carotenoids was synthesised by the CMIFS 119 strain (195.96 μg g^−1^), and in the remaining ones their content ranged from 141.14 (CMIFS 113) to 185.7 μg g^−1^ (CMIFS 116). The *C. capitatum* CMIFS 119 isolate also showed the highest volumetric yield of carotenoid biosynthesis (2.95 mg L^−1^) among all strains belonging to this genus. For strains CMIFS 104, 113 and 116, lower yields of 1.51 to 1.79 mg L^−1^ were obtained. Vysoka et al., (2023) tested various strains of red yeast for the ability to biosynthesise carotenoids, including *C. infirmominiatum* CCY 17-18-4. This strain, during cultivation in a medium with glucose and the addition of KNO_3_, KH_2_PO_4_ and MgSO_4_·7H_2_O, produced much less carotenoids (7 μg g^−1^) than the strains tested in this work.

The content of carotenoids in the yeast biomass belonging to the species *R. colostri* did not exceed 250 μg g^−1^ and ranged from 190.75 (CMIFS 106) to 228.82 μg g^−1^ (CMIFS 118). The work of Li et al. (Li et al. 2022a) showed that the yeast belonging to *R. colostri* can synthesise much higher amounts of carotenoids at low temperatures. After 5 days of cultivation at 16 °C, the volumetric yield of carotenoid biosynthesis increased to 29.016 mg L^−1^ compared to the control culture (17.147 mg L^−1^) conducted at 25 °C.

Yeast belonging to the species *P. rhodozyma* (CMIFS 102, 110, 114) synthesised carotenoids ranging from 85.63 to 131.08 μg g^−1^. After taking into account the value of biomass yields, the volumetric yields of biosynthesis were low and ranged from 0.33 to 0.55 mg L^−1^. Due to the ability to produce carotenoids, *P. rhodozyma* yeast is used industrially as a feed additive for farmed fish and marine crustaceans (Johnson 2013). In addition, carotenoids have a positive effect on improving the health of animals (Elwan et al. 2019), and the addition of yeast biomass to food intended for poultry contributes to the improvement of the colour of egg yolks, which makes them more attractive to the consumer (Zhu et al. 2021).

One of the three isolates of *C. psychroaquaticum* (CMIFS 107) was also characterised by a high content of carotenoids in biomass (302.97 μg g^−1^). The other two strains synthesised these compounds at much lower levels. The volume yield of carotenoid biosynthesis was higher than 2 mg L^−1^ only in the CMIFS 112 strain due to the high yield of biomass. In the work of Chreptowicz et al. (2019), the yeast *C. psychroaquaticum* WUT 117 synthesised carotenoids with different efficiency, depending on the initial C/N molar ratio. The content of carotenoids ranged from 37 (C/N = 60/1) to as much as 530 μg g^−1^ (C/N = 20/1). The highest yield (3.15 mg L^−1^) was also obtained in a medium with C/N = 20/1.

For the yeast *B. aurantiaca* CMIFS 105, the content of carotenoids was found to be 170.77 μg g^−1^. Almost 3 times higher (504.4 μg g^−1^) amounts of these compounds were obtained by Kim et al. (2011) while cultivating *B. aurantiaca* K-505 at 25 °C in glucose, yeast extract, peptone and ammonium sulfate medium at pH 1.0. The yeast *S. coprosmae* CMFS 108 also produced more than 100 μg g^−1^ of carotenoid pigments, and the volumetric yield was 1.87 mg L^−1^.

### β-carotene

All tested yeast strains synthesised β-carotene in the medium with glucose, peptone and yeast extract. The highest percentage content of this compound was found in two strains of yeast *P. rhodozyma,* and it was 44.80% (CMIFS 102) and 46.27% (CMIFS 114). Among the eight tested yeast strains of the genus *Rhodotorula*, only *R. babjevae* CMIFS 109 and *R. mucilaginosa* CMIFS 101 produced over 10% of β-carotene. For the rest of the isolates, its content ranged from 3.75 to 9.72%. The percentages of individual carotenoids significantly depend on the composition of the medium and cultivation conditions. Cheng and Yang (2016) tested the effect of the initial pH of the medium on the process of carotenoid biosynthesis by the *R. mucilaginosa* F-1 strain. The highest content of β-carotene (41.4%) was found after cultivation in a medium with an initial pH of 4.0. In turn, Zhao and Li (2022) checked the effect of different temperatures (16, 25 and 32 °C) of the culture on the profile of carotenoids synthesised by the yeast *R. glutinis* ZHK. It was found that yeast produced the most β-carotene (49.77%) at 25 °C, while much less of this pigment was determined in biomass grown both at low and high temperatures, 22.47 and 25.79%, respectively.

A large amount (31.53%) of β-carotene, compared to other carotenoids, was also synthesised by the strain *C. psychroaquaticum* CMIFS 112. The other two strains of this species (CMIFS 107 and 111) produced 3.05 and 2.33%, respectively. *Cystofilobasidium* yeasts were characterised by a very different percentage of β-carotene, ranging from 4.50 (CMIFS 119) to 20.13% (CMIFS 116). During a 72-h cultivation in a medium with glucose, yeast extract, peptone and the addition of mineral salts, the yeast *C. capitatum* VKPM Y-3202 synthesised significantly more β-carotene, and its percentage in the total pool of carotenoids was 63% (Yurkov et al. 2008).

In the case of yeast *B. aurantiaca* CMIFS 105, the percentage of β-carotene content did not exceed 15%. Most strains of *R. colostri* were characterised by a low content of β-carotene in the total pool of carotenoids (1.38–5.86%), except for the CMIFS 118 strain, which synthesised 11.72% of this compound. Slightly more β-carotene (15.70%) was produced by the yeast *Rhodosporididiobolus odoratus* when cultured in a medium with glucose (Zhao et al. 2023). The *S. coprosmae* CMIFS 108 strain produced only 2.52% of β-carotene.

### Torulene

Different percentages of torulene content in the total pool of yeast carotenoids were found. The largest amount of this compound (59.25%) was synthesised by the strain *C. psychroaquaticum* CMIFS 112.

The other two strains of this species (CMIFS 107 and 111) produced 20.77 and 17.13% of torulene, respectively. An equally high amount of torulene as the CMIFS 112 strain was also synthesised by one of the four strains of *C. capitatum* (CMIFS 113), and it was 55.44%. In the remaining tested strains of this species, the percentage of torulene ranged from 11.08 to 40.07%. After 72 h of culturing the yeast *C. capitatum* VKPM Y-3202, the content of torulene was equal to 17% (Yurkov et al. 2008).

In the case of yeasts of the genus *Rhodotorula*, the content of torulene in the biomass varied depending on the species. Strains belonging to *R. mucilaginosa* synthesised from 25.19 to 46.96%, *R. babjevae* 6.12%, and *R. glutinis* only 1.11%. The production of this carotenoid in yeast cells can be stimulated by the addition of stressors to the medium. It was shown (Elfeky et al. 2019) that after adding 0.7 mM Al_2_(SO_4_)_3_ to the medium, the yeast *R. glutinis* AS 2.703 synthesised as much as 98% of torulene in the total pool of carotenoids.

Over 30% of torulene was also produced by the yeast *B. aurantiaca* CMIFS 110. The strains of *R. colostri* during cultivation produced a varied amount of this carotenoid, which ranged from 15.19 to 26.02% of the total pool of carotenoids. The yeast *R. odoratus* XQR during 72-h cultivation at 20 °C in the medium with dextrose synthesised much more torulene − 77.76% (Zhao et al. 2023). A low content of torulene (14.45%) compared to other carotenoids was found in the yeast *S. coprosmae* CMIFS 108. *Phaffia rhodozyma* was characterised by a very low percentage of torulene (5.48–10.89%) in the total pool of carotenoids produced.

### Torularhodin

The content of torularhodin in the biomass of the tested yeast strains was the highest in comparison with other carotenoids. Among all yeasts, the greatest amount (86.82%) of this dye was synthesised by the *R. glutinis* strain (CMIFS 103). In turn, *R. babjevae* (CMIFS 109) produced 10% less torularhodin. In a study by Peng et al. (2021), when the yeast *R. babjevae* 05-775 was cultivated in a GMY medium with glycerol (40 g/L), the accumulation of torularhodin was over 90% of the total carotenoid production. The percentage of torularhodin in the total pool of carotenoids synthesised by the yeast species *R. mucilaginosa* was very diverse. The greatest amount of this dye was produced by strains CMIFS 099 (69.08%) and CMIFS 100 (66.89%).

*S. coprosmae* CMIFS 108 also contained more than 80% of torularhodin in the total pool of carotenoids. Equally high amounts of this compound were found for two strains of *R. colostri*: 79.99% (CMIFS 117) and 76.50% (CMIFS 115). The other two strains of this species produced significantly less of this compound: 60.3 and 61.97% for CMIFS 118 and 106, respectively. Another strain belonging to the genus *R. odoratus* XQR, in the study by Zhao et al. (2023), produced only 6.54% of torularhodin on the glucose medium, which is even 10 times less.

*C. psychroaquaticum* strains produced a very diverse amount of torularhodin, from as little as 6.12% (CMIFS 112) to as much as 77.48% for the CMIFS 111 strain. *Cystofilobasidium* yeasts were characterised by a low ability to biosynthesise torularhodin. Its percentage share ranged from 4.46 to 23.05%. The exception was *C. capitatum* strain CMIFS 119, which synthesised 66.56% of this dye. Torularhodin in yeast *B. aurantiaca* (CMIFS 108) accounted for almost half (45.19%) of all carotenoids produced. No presence of this carotenoid was found in the yeast biomass of *P. rhodozyma*.

### γ-carotene

The content of γ-carotene was below 10% for most of the tested yeast strains. The exception was the strain *C. infirmominiatum* CMIFS 116, which produced as much as 45.75% of this compound. Other strains belonging to this genus produced a small amount of γ-carotene ranging from 0.97 to 1.89%. A high content of γ-carotene in the cell biomass compared to other *Rhodotorula* yeasts was found in the *R. glutinis* CMIFS 103 strain, which synthesised 9.24% of this pigment. The *R. babjevae* CMIFS 109 isolate produced only 1.98% of γ-carotene. *R. mucilaginosa* strains synthesised γ-carotene ranging from 1.02 to 1.75% of the total carotenoid pool. The content of γ-carotene in the yeast biomass of *P. rhodozyma* was varied and amounted to 4.5% for the CMIFS 110 strain, 5.01% for CMIFS 114 and 8.74% for CMIFS 102. In the case of the tested yeasts of the species *C. psychroaquaticum*, *B. aurantiaca*, *S. coprosmae* and *R. colostri*, the share of γ-carotene did not exceed 4.10% in the total pool of carotenoids.

### Astaxanthin

Astaxanthin was produced only by *P. rhodozyma* yeast. The CMIFS 110 and 114 strains showed the synthesis of this carotenoid at a practically identical level, 35.29 and 35.26%, respectively, while in the biomass of the CMIFS 102 strain, there was less astaxanthin (25.32%). Li et al. (2023) obtained as much as 79% of the astaxanthin in the yeast biomass of *P. rhodozyma* CBS 6938, and this value increased to 88.3% by inducing oxidative stress in cells by adding plasma-activated water to the medium. Schewe et al. (2017) also showed the effect of lowering the pH from 5.5 to 3.5 in the stationary phase on increasing the percentage of astaxanthin from about 60 to 70% in the yeast biomass of *P. rhodozyma* AXJ-20.

### Biosynthesis of exopolysaccharides

**Fig. 3:**
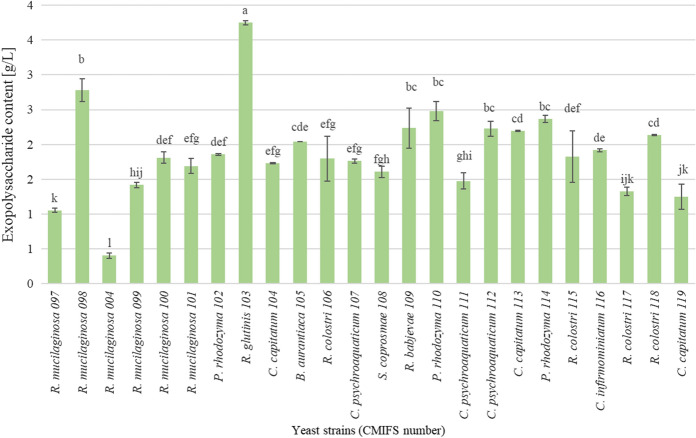
Content of exopolysaccharides in post-culture media obtained after 96 h of red yeast cultivation. Indexes a.b.c… means homogeneous groups determined by Tukey’s test (one-way analysis of variance)

Based on the obtained results, it was found that the tested yeast strains are not efficient producers of extracellular polysaccharides (Fig. 3). Their content was the highest in the post-culture medium of the *R. glutinis* CMIFS 103 strain and amounted to 3.75 g L^−1^. In the studies of Ramirez and Ramirez (2015), in the supernatant of yeast *R. glutinis*, a significantly lower amount of exopolysaccharides was found, ranging from 0.58 to 0.80 g L^−1^, depending on the composition and pH of the medium, as well as the cultivation time. Strains belonging to the species *R. mucilaginosa* produced a varied content of these compounds in the range from 0.40 (CMIFS 004) to 2.78 g L^−1^ (CMIFS 098).

Li et al. (2020) studied the effect of carbon source, nitrogen source, temperature and culture time on the production efficiency of extracellular polysaccharides. It was shown that the yeast *R. mucilaginosa* YL-1 produced the highest amount (15.1 g L^−1^) of exopolysaccharides after 96 h of cultivation at 24 °C in a medium with glucose (50 g L^−1^) and yeast extract (10 g L^−1^). Hamidi and co-authors obtained an even higher content (28.5 g L^−1^) of these compounds during a 5-day cultivation of another *R. mucilaginosa* GUMS16 strain at 25 °C (Hamidi et al. 2020). The yeast *R. babjevae* CMIFS 109 produced 2.24 g L^−1^ of exopolysaccharides. While studying another strain of this species, Seveiri and co-authors obtained a lower amount of exopolysaccharides (1.6 g L^−1^) in the medium with glucose, (NH_4_)_2_SO_4,_ KH_2_PO_4, _MgSO_4_·7H_2_O, NaCl, CaCl_2_·2H_2_O and yeast extract (Seveiri et al. 2020).

Among the yeasts of the genus *Cystofilobasidium*, the highest amount of exopolysaccharides was produced by the strain CMIFS 113 (2.20 g L^−1^). Above 2 g L^−1^ of exopolysaccharides was synthesised by only one (CMIFS 118) of four strains of *R. colostri*. The total exopolysaccharides content of the *B. aurantiaca* CMIFS 105 yeast supernatant was 2.04 g L^−1^. After cultivation *S. coprosmae* CMIFS 108, a low amount of exopolysaccharides was found in the post-culture medium (1.61 g L^−1^). Two strains of *P. rhodozyma* (CMIFS 110 and 114) synthesised EPS at the same high level compared to the other tested yeasts, 2.48 and 2.37 g L^−1^, respectively. Significantly lower (1.86 g L^−1^) content of these compounds was found during the cultivation of *C. psychroaquaticum* strains. The studied strains of *C. psychroaquaticum* were characterised by a diverse biosynthesis of exopolysaccharides, which ranged from 1.48 (CMIFS 111) to 2.23 g L^−1^ (CMIFS 112). Rusinova-Videva and co-authors also used sucrose as a carbon source in the medium and showed a similar content (2.10 g L^−1^) of exopolysaccharides after 72 h of cultivation of the *Cystobasidium ongulense* strain (Rusinova-Videva et al. 2023).

So far, in the literature, in terms of the synthesis of exopolysaccharides, the best-described yeasts belong to such genera as *Candida, Pichia, Aureobasidium, Tremella*, and among red yeasts, various species of *Rhodotorula, Sporobolomyces* and *Cystobasidium* (Rahbar Saadat et al. 2021). The use of the remaining red yeast to produce these compounds has not yet been described.

### Lipid biosynthesis

**Fig. 4:**
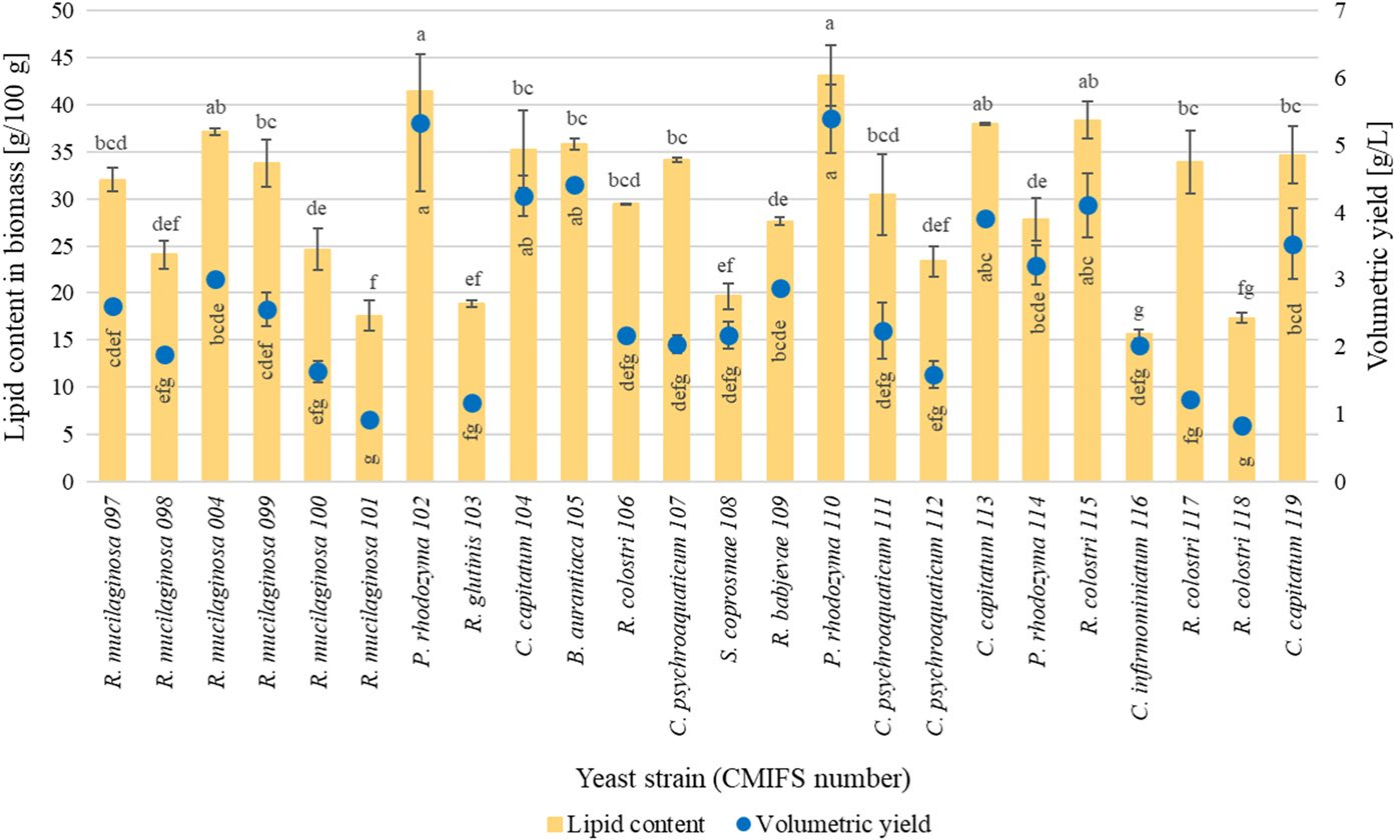
Total lipid content and their volumetric yield of biosynthesis after 96 h of cultivation. Indexes a.b.c… means homogeneous groups determined by Tukey’s test (one-way analysis of variance)

In order to determine the ability of the tested yeast strains for lipid biosynthesis, a medium with an initial molar ratio of C/N = 70/1 was used. A high value of this parameter stimulates the de novo lipid biosynthesis by oleaginous yeast strains (Elfeky et al. 2019; Lopes et al. 2020). All tested yeast strains synthesised intracellular lipids (Fig. 4). It was shown that two *P. rhodozyma* isolates (CMIFS 102 and 110) produced microbial lipids at the same high level, respectively 41.41 and 43.14 g 100 g^−1^. Also, for these two strains, the highest biosynthetic volumetric yield was found (5.33–5.40 g L^−1^). A lower amount of lipids in the biomass (27.86 g 100 g^−1^) and a volumetric yield (3.22 g L^−1^) were obtained when cultivating the CMIFS 114 strain. Xiao et al. (2015) investigated the relationship between intracellular lipid anabolism and carotenoid production in the yeast *P. rhodozyma*. They showed that in the biomass of the mutant strain JMU-MVP14 overproducing astaxanthin, the lipid content was the lowest (approx. 12 g 100 g^−1^). In turn, in the biomass of the strain that produced 10 times less astaxanthin, the lipid content increased to about 25 g 100 g^−1^.

The yeast belonging to *R. mucilaginosa* produced a varied amount of lipids, which ranged from 17.59 (CMIFS 101) to 37.14 g 100 g^−1^ (CMIFS 004). Volumetric yield also varied widely (from 0.92 to 3.01 g L^−1^). Liang et al. (2021) tested the effect of temperature, carbon source, nitrogen source and C/N ratio on lipid production by the *R. mucilaginosa* LP-2 strain. The highest accumulation of lipids by this yeast was found at 33 °C (6.48 g L^−1^), the best source of carbon was pure glycerol (7.12 g L^−1^), and the best source of nitrogen was yeast extract (6.1 g L^−1^). *R. babjevae* CMIFS 109 produced 27.62 g 100 g^−1^ of lipids, while *R. glutinis* CMIFS 103 produced only 18.85 g 100 g^−1^. In a study by Peng et al. (2021), when the yeast *R. babjevae* 05-775 was cultivated in a GMY medium with yeast extract (3 g L^−1^) and glycerol (40 g L^−1^), the accumulation of lipids after 144 h was only 24.39 g 100 g^−1^.

The yeast C. *capitatum* CMIFS 104, 113 and 119 synthesised a similar amount of lipids, ranging from 34.68 to 38.01 g 100 g^−1^, while *C. infirmominiatum* CMIFS 116 produced only 15.66% of lipids in the dry cell substance. Also, the volumetric yield values were varied and for *C. capitatum* they were 3.40–4.24 g L^−1^, and for *C. infirmominiatum,* only 2.03 g L^−1^. In the work of Petrik et al. (2013), the *C. capitatum* CCY 10-1-1 strain synthesised 22.6 g 100 g^−1^ of lipids using waste glycerol after the production of biofuels.

A high content of lipids in biomass was found in two (CMIFS 115 and 117) out of four *R. colostri* strains, which produced 38.39 and 33.91 g 100 g^−1^, respectively. The remaining two strains synthesised significantly less of these compounds. The volumetric yield of more than 4 g L^−1^ of lipids was obtained only for one strain—CMIFS 115. The work of Poontawee et al. (2018) optimised the production of lipids by *Rhodosporidiobolus fluvialis* DMKU-SP314 in a medium consisting of sugar cane and glycerol derived from biodiesel production. After 240 h of cultivation at 28 °C, the yeast biomass contained 75% of lipids.

Above 30 g 100 g^−1^ of lipids were synthesised by two (CMIFS 107 and 111) strains of *C. psychroaquaticum*, the third isolate (CMIFS 112) produced much less of these compounds (23.35 g 100 g^−1^). Vyas and Chhabra (2017) studied the effect of different carbon sources on lipids production by the *Cystobasidium oligophagum* JRC1 strain. The percentage of lipids was highest for glycerol (42.04 g 100 g^−1^), next by starch (41.54 g 100 g^−1^) and glucose (39.44 g 100 g^−1^), and the lowest for lactose (21.91 g 100 g^−1^) and sucrose (21.72 g 100 g^−1^).

Two (CMIFS 107 and 111) of the three strains of *C. psychroaquaticum* synthesised lipids at the same level (2.04 and 2.25 g L^−1^). A lower (1.6 g L^−1^) yield was obtained when cultivating the third strain (CMIFS 112). During the research of Chreptowicz et al. (2021), the yeast *C. psychroaquaticum* WUT 117 synthesised lipids with varying efficiency, depending on the carbon source used. The highest yield was achieved in the medium with glucose (15.11 g L^−1^), glycerol (12.34 g L^−1^), sucrose (12.08 g L^−1^) and xylose (9.88 g L^−1^).

The total lipid content in the yeast biomass of *B. aurantiaca* CMIFS 105 was 35.82 g 100 g^−1^, and the volumetric yield was 4.42 g L^−1^. In the work of Petrik et al. (2013), the yeast *B. aurantiaca* CCY 20-9-7 showed a greater production of lipids (20%) from glycerol than from glucose (synthes. 13%). After culturing the yeast *S. coprosmae* CMIFS 108, a low amount of lipids in the biomass was found, equal to 19.68 g 100 g^−1^, which also resulted in a low volumetric yield (2.17 g L^−1^).

### Fatty acids of red yeast

**Fig. 5:**
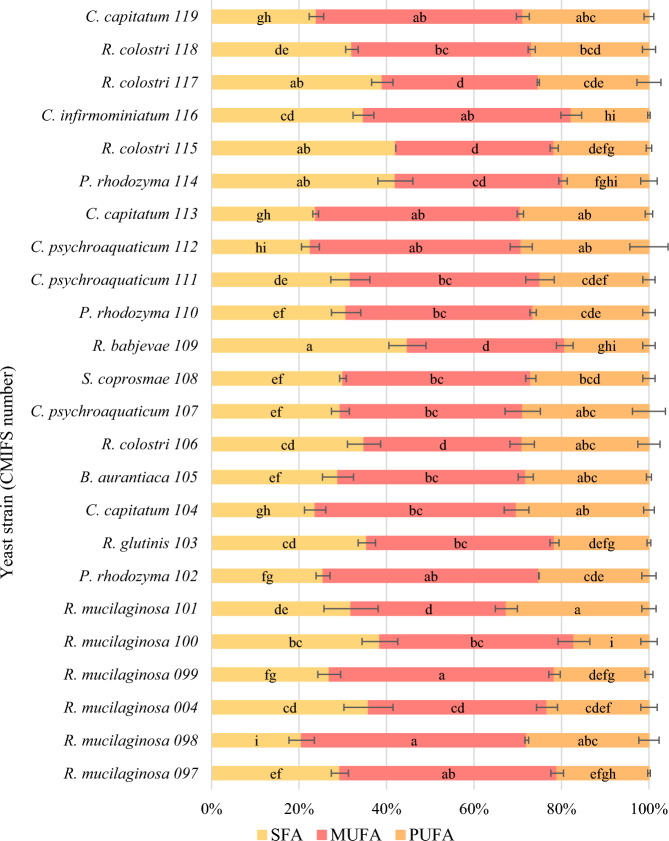
Profile of fatty acids (*SFA* saturated fatty acids, *MUFA* monounsaturated fatty acids, *PUFA* polyunsaturated fatty acids) in the cell biomass of the tested red yeast strains after 96 h of cultivation. Indexes a.b.c… means homogeneous groups determined by Tukey’s test (one-way analysis of variance)

Taking into account all yeast strains, the highest content (44.79%) of saturated fatty acids was found in *R. babjevae* CMIFS 109 (Fig. 5). It was mainly palmitic acid (20.46%) and stearic acid (17.15%). Among the monounsaturated fatty acids (35.96%) produced by this strain, oleic acid dominated (35.0%). It was mainly palmitic acid (20.46%) and stearic acid (17.15%). Among the monounsaturated fatty acids (35.96%) produced by this strain, oleic acid dominated (35.0%).

The content of PUFA in the biomass of *R. babjevae* (CMIFS 109) was much lower and amounted to 19.25%. This strain was also the only one among all tested yeasts to produce slightly more linolenic acid (9.92%) than linoleic acid (9.33%). Peng et al. (2021), during a 120-h culture of yeast *R. babjevae* 05-775 in a medium with a limited amount of nitrogen and glycerol, showed a slightly higher content of PUFA (24.2%). Other ratios of linoleic acid (17.4%) to linolenic acid (3.8%) were also found (Table 4).

**Table 4:** Percentages of individual fatty acids after 96 h of red yeast cultivation

Yeast strain (CMIFS number)	Palmitic acid (%)	Stearic acid (%)	Oleic acid (%)	Linoleic acid (%)	Linolenic acid (%)
*R. mucilaginosa* 097	16.30	6.99	48.47	17.22	3.75
*R. mucilaginosa* 098	3.76	11.62	50.76	18.70	9.22
*R. mucilaginosa* 004	15.91	12.30	39.81	14.31	8.99
*R. mucilaginosa* 099	14.87	6.27	50.66	17.50	4.10
*R. mucilaginosa* 100	19.30	9.82	39.94	13.63	3.52
*R. mucilaginosa* 101	13.45	10.52	34.91	25.55	7.04
*P. rhodozyma* 102	15.73	5.96	49.32	24.09	1.07
*R. glutinis* 103	25.23	6.89	41.50	16.20	5.39
*C. capitatum* 104	17.66	4.78	46.20	28.87	1.63
*B. aurantiaca* 105	21.47	5.71	41.64	26.69	1.48
*R. colostri* 106	23.82	6.76	36.13	23.86	5.13
*C. psychroaquaticum* 107	17.02	8.73	41.70	27.28	1.57
*S. coprosmae* 108	24.71	3.75	42.38	24.84	2.17
*R. babjevae* 109	20.46	17.15	35.0	9.33	9.92
*P. rhodozyma* 110	18.37	7.67	42.74	25.85	0.64
*C. psychroaquaticum* 111	20.95	5.19	43.36	23.16	1.74
*C. psychroaquaticum* 112	14.06	4.91	48.19	26.38	2.82
*C. capitatum* 113	17.18	4.74	46.46	27.86	1.53
*P. rhodozyma* 114	19.66	5.19	38.32	18.21	1.44
*R. colostri* 115	26.99	5.48	36.22	18.65	3.01
*C. infirmominiatum* 116	23.90	5.25	47.50	16.15	1.60
*R. colostri* 117	27.72	2.6	35.68	22.48	2.80
*R. colostri* 118	23.15	5.64	41.08	23.91	2.90
*C. capitatum* 119	18.22	3.97	47.17	27.14	1.69

*R. glutinis* (CMIFS 103) produced about 10% less SFA than *R. babjevae* (CMIFS 109), and the majority (25.23%) was palmitic acid. Significantly more oleic acid was synthesised, and the total MUFA content was 42.89%. *R. glutinis* strain CMIFS 103 produced 21.60% of polyunsaturated fatty acids, of which 16.2% was linoleic acid and 5.39% linolenic acid. When *R. glutinis* LOCKR13 was cultivated in a medium with glycerol at 20 °C by Kot et al. (2019b), synthesised 29.7% PUFA. Low temperature was also found to stimulate lipid biosynthesis by yeast.

Strains belonging to *R. mucilaginosa* showed a varied SFA content ranging from 20.6 (CMIFS 098) to 38.5% (CMIFS 100). Only in the case of the CMIFS 098 isolate, a higher content of stearic acid (11.62%) than palmitic acid (3.76%) was found. The opposite relationship was observed for the other strains. The MUFA content in yeast *R. mucilaginosa* lipids was the highest. Most of these acids (approx. 51%) were synthesised by CMIFS 098 and 099 strains. The remaining strains were characterized by a very diverse amount of MUFA, from only 35.51 (CMIFS 101) to 49.68% (CMIFS 097). The content of PUFA in the lipids of *R. mucilaginosa* strains ranged from 17.15 (CMIFS 100) to 32.59% (CMIFS 101). The majority of PUFA in the biomass of all strains was linoleic acid, its amount ranging from 13.63 (CMIFS 100) to 25.55% (CMIFS 101). The amount of linolenic acid was much lower and did not exceed 5% for strains CMIFS 097, 099 and 100, and 10% for the remaining three (CMIFS 004, 098 and 101). Liang et al. (2021) observed the relationship that the higher the C/N ratio in the culture medium, the yeast *R. mucilaginosa* LP-2 synthesizes more PUFA. At the lowest C/N ratio (25), polyunsaturated fatty acids were not detectable, while at the higher ratio (85), their content was at the level of 15.5%.

Two strains of *R. colostri* (CMIFS 115 and 117) synthesised saturated fatty acids at the same level, 42.12 and 39.04%, respectively. The other two strains produced significantly less SFA − 34.88% (CMIFS 106) and 32.11% for CMIFS 118. In the lipids of all yeast *R. colostri*, palmitic acid constituted the majority (23.15–27.72%) of saturated acids, while the content of stearic acid was low and ranged from 2.60 to 6.76%. Only one strain (CMIFS 118) contained above 40% MUFA in the lipid fraction. The remaining strains synthesised significantly less of these fatty acids: between 35.68 (CMIFS 117) and 36.23% for CMIFS 106. Above 25% PUFA was synthesised by three strains of yeast *R. colostri* (CMIFS 106, 117, 118). Strain CMIFS 115 produced slightly less of these compounds (21.66%). The highest amount of linoleic acid was obtained after culturing the CMIFS 118 strain (23.91%) and the least for the CMIFS 115 strain (18.65%). The CMIFS 106 isolate showed the best production of linolenic acid (5.13%). In the yeast biomass of *R. fluvialis* DMKU-SP314 obtained by Poontawee et al. (2018), a similar content of PUFA was found, ranging from 21.5 to 26.8% depending on culture parameters such as time, aeration or pH. The vast majority (17.9–22.7%) was linoleic acid.

In the case of *P. rhodozyma*, the SFA content was very diverse and ranged from 25.52 (CMIFS 102) to 42.04% (CMIFS 114). In all three tested strains, the amount of palmitic acid was similar and accounted for more than half of all saturated fatty acids, and stearic acid was much less (5.19–7.67%). Also, the MUFA content was varied and ranged from 38.32 (CMIFS 114) to as much as 49.33% (CMIFS 102). During the cultivation of *P. rhodozyma* CCY 77-1 for 96 h in a medium with glucose and the addition of KNO_3_, KH_2_PO_4_ and MgSO_4_·7H_2_O, a similar content of MUFA (42%) was found (Vysoka et al. 2023). In the work of Mussagy et al. (2022), after the extraction of lipids from the biomass of *P. rhodozyma* NRRL Y-17268, as much as 66.18% of monounsaturated fatty acids were obtained, with the main part being oleic acid (64.2%). Two *P. rhodozyma* yeast strains (CMIFS 102 and 110) were characterised by the same level of PUFA biosynthesis (25.17–26.81%). The CMIFS 114 strain produced a lower amount of these compounds, namely 19.65%. All strains synthesised mainly linoleic acid. The use of a different medium during the cultivation of *P. rhodozyma* CCY 77-1 allowed Vysoka et al. (2023) to obtain as much as 38% PUFA.

The content of saturated fatty acids for all strains of *C. capitatum* (CMIFS 104, 113 and 119) was practically identical and ranged from 23.82 to 24.00%. Each of these strains accumulated more than 17% of palmitic acid and less than 5% of stearic acid. Above 30% SFA was produced only by the *C. infirmominiatum* (CMIFS 116) because this strain synthesised more of both palmitic acid (23.90%) and stearic acid (5.25%). Statistical analysis showed that all tested strains of the genus *Cystofilobasidium* produce monounsaturated fatty acids at the same level, ranging from 46.40 (CMIFS 104) to 47.50% (CMIFS 116). For the yeast *C. infirmominiatum* in the study conducted by Vysoka et al. (2023), a slightly lower (36%) amount of MUFA was found compared to the strain tested in this work. PUFA content varied depending on the species. Strains belonging to *C. capitatum* synthesised more (28.83–30.50%), and *C. infirmominiatum* only 17.75%. The vast majority of PUFA was linoleic acid—from 16.15 (CMIFS 116) to 28.87% for the CMIFS 104 strain. Linolenic acid was very low, and its value did not exceed 2%.

The highest percentage of saturated fatty acids among yeast *C. psychroaquaticum* was found during the cultivation of strains CMIFS 111 (31.74%) and CMIFS 107 (29.46%), and the CMIFS 112 isolate produced much less (22.61%). In the lipids of these yeasts, the percentage of palmitic acid ranged from 14.06 to 20.95%. Vyas and Chhabra (2017) showed a higher SFA content of 31.33% in the yeast *C. oligophagum* JRC1 lipids obtained after cultivation in a glucose medium. A very large part of this was palmitic acid (26.02%), while stearic acid was only 4.15%. *C. psychroaquaticum* strain CMIFS 112 was characterised by the highest content of monounsaturated fatty acids (48.19%). The other two strains produced less (41.70–43.37%). Another strain belonging to the same genus, *C. oligophagum* JRC1 synthesised MUFA at a similar level of 43.22% (Vyas and Chhabra 2017). For all tested strains of *C. psychroaquaticum*, a similar amount of polyunsaturated fatty acids was found, which ranged from 24.90 (CMIFS 111) to 29.21% (CMIFS 112). In the biomass of these yeasts, the percentage of linoleic acid accounted for almost all of the PUFA content, and linolenic acid was low, from 1.57 to 2.82%. After cultivating *Cystobasidium* yeast in a glucose medium, Vyas and Chhabra (2017) showed that polyunsaturated fatty acids were present in the amount of 25.05%. The content of linoleic acid was found to be 24.22%, and linolenic acid only 0.83%.

The average content of saturated fatty acids (30.06%) compared to the other tested yeasts was synthesised by the *S. coprosmae* isolate (CMIFS 108). The vast majority (24.71%) was palmitic acid, while stearic acid was scarce (3.75%). A similar amount of all SFA (28.92%), palmitic acid (21.47%) and stearic acid (5.71%) were also found in yeast *B. aurantiaca* (CMIFS 105). The content of monounsaturated fatty acids after culturing the yeasts *S. coprosmae* and *B. aurantiaca* was at the same level and amounted to 42.94 and 42.91%, respectively. Both of these strains produced mainly oleic acid. Compared to other strains, yeast *B. aurantiaca* and *S. coprosmae* produced a high amount of polyunsaturated fatty acids, which was 28.18 and 27.01%, respectively. A relatively high amount of linoleic acid (26.69%) was also found in the biomass of the CMIFS 105 strain.

### Enzyme biosynthesis

Of all the tested strains, thirteen showed no ability to synthesise lipolytic enzymes when cultivated on an agar medium with tributyrin (Table 5). These were the strains: *R. mucilaginosa* CMIFS 098, *C. capitatum* CMIFS 113, *C. infirmominiatum* CMIFS 116 and all strains belonging to *P. rhodozyma* (CMIFS 102, 110 and 114), *R. colostri* (CMIFS 106, 115, 117, 118) and *C. psychroaquaticum* (CMIFS 107, 111, 112). Interestingly, *C. psychroaquaticum* strains isolated by Chreptowicz et al. (2021) showed high lipolytic activity. In the medium with olive oil and Tween 80, one of the isolates produced 346.69 U mL^−1^ of lipases at 20 °C after 96 h of cultivation, and the other as much as 426.81 U mL^−1^ at 15 °C after 72 h. Enzyme unit (U) was defined as the amount of enzyme that resulted in the release of 1 μmol of p-NPP per millilitre of medium per minute under the conditions tested.

**Table 5:** Results of red yeast enzymatic activity determination by plate method (− indicates a negative result; + positive result (zone diameter less than 1 cm); ++ positive result (zone diameter 1–2 cm); +++ positive result (zone diameter greater than 2 cm))

Yeast strain (CMIFS number)	Cellulolytic activity	Amylolytic activity	Proteolytic activity	Lipolytic activity
*R. mucilaginosa* 097	−	−	−	+
*R. mucilaginosa* 098	−	−	−	−
*R. mucilaginosa* 004	−	+	−	+
*R. mucilaginosa* 099	−	++	−	+
*R. mucilaginosa* 100	−	+	−	+
*R. mucilaginosa* 101	−	++	−	++
*P. rhodozyma* 102	−	−	−	−
*R. glutinis* 103	−	−	−	+++
*C. capitatum* 104	−	+	++	++
*B. aurantiaca* 105	−	−	−	++
*R. colostri* 106	−	−	−	−
*C. psychroaquaticum* 107	−	−	−	−
*S. coprosmae* 108	−	+++	++	+
*R. babjevae* 109	−	−	−	++
*P. rhodozyma* 110	−	−	−	−
*C. psychroaquaticum* 111	−	−	−	−
*C. psychroaquaticum* 112	−	−	−	−
*C. capitatum* 113	−	+	−	−
*P. rhodozyma* 114	−	−	−	−
*R. colostri* 115	−	−	−	−
*C. infirmominiatum* 116	−	+	−	−
*R. colostri* 117	−	−	−	−
*R. colostri* 118	−	−	−	−
*C. capitatum* 119	−	+	−	++

The lipolytic activity of eleven strains was varied, and the largest zone of tritutyrin decay was noted for the *R. glutinis* CMIFS 103 strain. Yeasts belonging to this species are described in the literature as efficient producers of extracellular lipolytic enzymes (Hatzinikolaou et al. 1999; Khayati and Alizadeh 2013; Taskin et al. 2016). For example, Taskin et al. (2016) studied the effect of temperature, initial pH of the medium and culture time on the production of lipases by the yeast *R. glutinis* HL25. One unit (U) of lipase activity was defined as the amount of enzyme (contained in 1 L of medium) that hydrolyses the conversion of 1 μmol p-NPP per minute of an experiment. The maximum lipase activity was obtained at a temperature of 20 °C (14.5 U L^−1^), a pH of 6.0 (14.5 U L^−1^) and 72 h of cultivation (75.2 U L^−1^) in a medium containing post-frying olive oil. In addition, they showed that the use of the method of immobilisation of these yeast cells has a positive effect on the synthesis of extracellular lipolytic enzymes.

In addition to the yeast *R. glutinis* CMIFS 103, strains belonging to *R. mucilaginosa* (CMIFS 097, 004, 099, 100, 101), *C. capitatum* (CMIFS 104 and 119), *B. aurantiaca* (CMIFS 105), *S. coprosmae* (CMIFS 108) and *R. babjevae* (CMIFS 109). The use of various lipase synthesis inductors during the cultivation of *R. mucilaginosa* MTCC-8737 allowed Chennupati et al. (2009) to obtain enzymes with different activities. One unit of lipase activity was defined as the amount of enzyme that resulted in the release of 1 μmol of p-nitrophenol per litre of medium per minute. The highest production of lipases was found after the application of soybean oil (29,589 U L^−1^) and olive oil (25,433 U L^−1^), and the lowest in the medium with coconut oil (5468 U L^−1^).

None of the tested yeast strains showed cellulolytic activity. Commercially, cellulases are obtained mainly from modified *Trichoderma reesei* mould strains. Red yeast *Rhodotorula mucilaginosa* has also been shown to be able to synthesise these enzymes in a carboxymethyl cellulose (CMC) medium (Parmar et al. 2023). Also, Rani et al. (2015) isolated a strain of *Rhodotorula glutinis* from decomposing vegetables, which produced β-glucosidase. These yeasts were cultured in various substrates in order to obtain the highest cellulolytic activity. It was observed that this strain synthesised the most cellulases when the carbon source was cellobiose (0.1%) and the nitrogen source was soybean meal. After a thorough analysis of this enzyme, it was also found that it shows maximum activity at 50 °C and at a pH of 6.0 to 6.5. In turn, Vyas and Chhabra (2017) isolated 4 strains of yeast *Cystobasidium oligophagum* from the soil, two of which showed cellulolytic activity on a medium with carboxymethylcellulose, yeast extract, KH_2_PO_4_, K_2_HPO_4_, MgSO_4_·7 H_2_O, (NH_4_)_2_SO_4_ and agar.

Of the twenty-four tested yeast strains, only nine produced amylolytic enzymes after 1 week of cultivation on a starch-peptone agar medium. These included four strains belonging to the species *R. mucilaginosa* (CMIFS 004, 099, 100, 101), the yeast *S. coprosmae* (CMIFS 108) and all strains identified as *Cystofilobasidium* (CMIFS 104, 113, 116 and 119). Carrasco et al. (2016) studied the effect of culture temperature and pH of the medium on the production of amylolytic enzymes by various yeast species, including *Rhodotorula* yeast. In the case of *Rhodotorula glacialis*, the highest amylolytic activity was found at lower temperatures (10–22 °C), at pH 5.4 and 6.2. Daskaya-Dikmen et al. (2018) studied red yeast strains isolated from environmental samples for the production of amylolytic enzymes at low temperatures. Both the yeast strains identified as *R. colostri* and *C. capitatum* showed amylolytic activity at 15 °C.

**Fig. 6:**
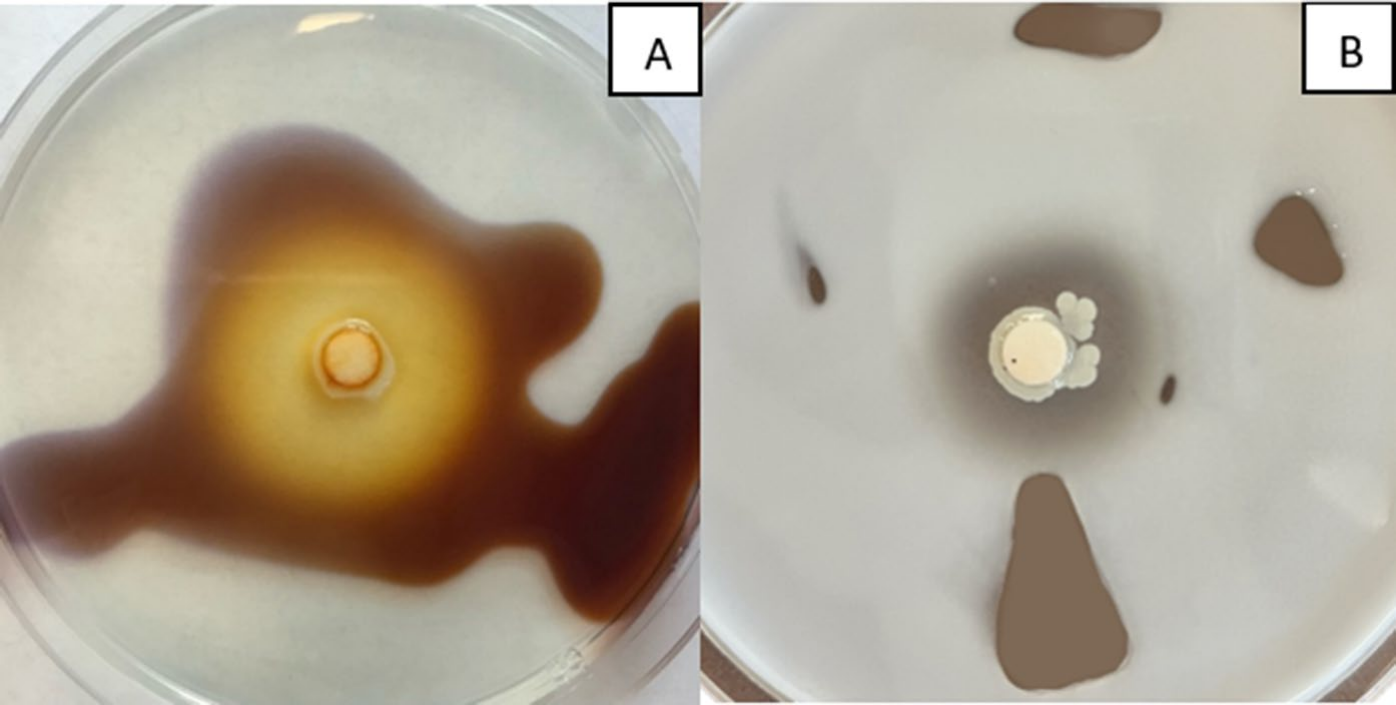
Photos of plates after cultivation *Symmetrospora coprosmae* CMIFS 108 on media with starch (**A**) and gelatin. The yellow zone of the medium after pouring Lugol’s solution on the plate indicates amylolytic activity (**A**), while the clear zone after dropping mercury (II) chloride solution on the gelatin medium indicates proteolytic properties (**B**)

Most of the tested yeast strains did not show the ability to synthesise proteolytic enzymes. The exceptions were one (CMIFS 104) of the four *Cystofilobasidium* isolates and the yeast *S. coprosmae* CMIFS 108 (Fig. 6). When the yeast *C. capitatum* was cultivated by Daskaya-Dikmen et al. (2018) at a lower temperature (15 °C), no proteolytic enzymes were found. *R. colostri* strains also showed no proteolytic activity in this study. Machado et al. (2022) isolated the yeast strain *R. mucilaginosa* from the Antarctic continent, which synthesised proteases with an activity of 124.9 U mL^−1^. Enzyme unit was defined as the amount of enzyme at which an increase in absorbance of 0.001 was found under the experimental conditions. Based on the obtained results, it was found that aeration and the amount of oxygen supplied were of great importance in the production of proteolytic enzymes during the cultivation of these yeasts. Other red yeasts are not described in the literature as microorganisms with high proteolytic activity.

### Biosynthesis of biosurfactants

Of the twenty-four yeast strains tested, only nine showed growth after 96 h in soybean oil medium. These were strains belonging to the species *R. mucilaginosa* (CMIFS 097, 004, 099, 100, 101 and 102), *R. glutinis* CMIFS 103, *R. babjevae* CMIFS 109, *S. coprosmae* CMIFS 108 and one of the strains *R. colostri* CMIFS 115. All three tests (Parafilm-M, Oil Displacement, and Phenol Sulfur) were performed without any positive results. This proved the inability of the tested strains to biosynthesis biosurfactants when cultured in a medium with soybean oil.

Bacteria belonging to the genus *Pseudomonas, Bacillus* or *Acinetobacter* are known to produce most groups of biosurfactants, including the most common glycolipids. Sophorolipids are the most studied and most promising class of extracellular glycolipids and are also produced by yeast. The presence of these compounds has been demonstrated during the cultivation of such species as: *Candida bombicola, Candida tropicalis, Starmerella bombicola, Candida apicola, Pichia anomala, Wickerhamiella domercqiae* and by the red yeast *Rhodotorula babjevae* and *Rhodotorula bogoriensis* (Solaiman et al. 2015; Kashif et al. 2022). The work by Guerfali et al. (2019) describes the secretion of emulsifying glycolipids by the *Rhodotorula babjevae* strain. It has been shown that the synthesis of biosurfactants by yeast is positively affected by limiting the amount of nitrogen in the substrate. Polyol lipids, on the other hand, are a class of extracellular glycolipids that include liamotins produced by the yeast-like fungus *Aureobasidium pullulans* and fatty acid polyol esters synthesised by various red yeast species: *Rhodosporidiobolus azoricus*, *Rhodotorula glutinis, Rhodotorula babjevae* or *Rhodotorula diobovata* (Garay et al. 2018). The other tested red yeast strains have not been characterised in the literature as producers of biosurfactants.

### Antagonist activity of red yeast

Among all tested yeasts, strains belonging to the genus *Rhodotorula* showed the best antagonistic activity against *Aspergillus niger* (Table 6). In their case, mould growth was reduced from 5 cm (control) to 1 cm (CMIFS 004) − 1.6 cm (CMIFS 103). A similar ability to inhibit the growth (1.8 cm) of *A. niger* was found for one of the strains of *C. capitatum* CMIFS 104. Other isolates of this genus showed less antimicrobial activity (2.5 cm—CMIFS 113) or did not show it at all (CMIFS 116 and 119). The yeast *R. colostri* was characterised by different antagonistic activity against A. niger because the growth zones of this mould ranged between 2 cm (CMIFS 118) and 5 cm (CMIFS 117). For all strains of *P. rhodozyma*, growth inhibition of *A. niger* was observed at a similar level (approx. 3 cm). Using only one isolate of *C. psychroaquaticum* (CMIFS 111) resulted in a smaller growth zone of *A. niger* (3.5 cm) than in the control, and the remaining strains showed no antagonistic activity. No such ability was found for the yeasts *S. coprosmae* and *B. aurantiaca*. The *A. niger* mould causes grape rot and causes serious economic losses to grapes worldwide. In vivo tests on grapes confirmed the activity of the red yeast *Sporidiobolus pararoseus* Y16 against *A. niger* (Li et al. 2017).

**Table 6:** Growth diameters of the mycelium of the moulds *Aspergillus niger, Botrytis cinerea, Penicillium expansum, Alternaria solani* and *Fusarium solani* on the medium inoculated with a suspension of the tested red yeast strains

Yeast strain (CMIFS number)	Growth diameter of *A. niger* (cm)	Growth diameter of *B. cinerea* (cm)	Growth diameter of *P. expansum* (cm)	Growth diameter of *A. solani* (cm)	Growth diameter of *F. solani* (cm)
*R. mucilaginosa* 097	1.1 ± 0.1^h^	0.9 ± 0.1^ef^	9.0 ± 0.0*	0.8 ± 0.1^ef^	3.4 ± 0.4^cd^
*R. mucilaginosa* 098	1.4 ± 0.2^fg^	0.7 ± 0.0^f^	9.0 ± 0.0*	0.6 ± 0.0^f^	3.0 ± 0.2^d^
*R. mucilaginosa* 004	1.0 ± 0.0^h^	0.8 ± 0.1^ef^	9.0 ± 0.0*	0.6 ± 0.0^f^	2.6 ± 0.1^e^
*R. mucilaginosa* 099	1.2 ± 0.1^gh^	0.8 ± 0.1^ef^	9.0 ± 0.0*	0.7 ± 0.0^f^	4.0 ± 0.2^b^
*R. mucilaginosa* 100	1.4 ± 0.2^fg^	1.3 ± 0.2^e^	9.0 ± 0.0*	0.7 ± 0.1^f^	3.3 ± 0.2^cd^
*R. mucilaginosa* 101	1.2 ± 0.2^gh^	0.9 ± 0.1^ef^	9.0 ± 0.0*	0.7 ± 0.1^f^	3.3 ± 0.3^cd^
*P. rhodozyma* 102	2.9 ± 0.3^c^	5.5 ± 0.3^c^	9.0 ± 0.0*	1.6 ± 0.2^bc^	4.0 ± 0.1^b^
*R. glutinis* 103	1.6 ± 0.2^f^	1.0 ± 0.1^e^	9.0 ± 0.0*	0.9 ± 0.0^e^	3.5 ± 0.2^c^
*C. capitatum* 104	1.8 ± 0.3^ef^	6.0 ± 0.2^c^	9.0 ± 0.0*	1.4 ± 0.1^cd^	4.7 ± 0.3^ab^
*B. aurantiaca* 105	5.0 ± 0.4^a^	8.0 ± 0.2^a^	9.0 ± 0.0*	2.0 ± 0.3^b^	5.3 ± 0.5^a^
*R. colostri* 106	3.7 ± 0.6^b^	7.5 ± 0.3^ab^	9.0 ± 0.0*	2.2 ± 0.2^b^	5.0 ± 0.3^a^
*C. psychroaquaticum* 107	5.0 ± 0.3^a^	8.0 ± 0.2^a^	9.0 ± 0.0*	2.0 ± 0.3^b^	5.3 ± 0.2^a^
*S. coprosmae* 108	5.0 ± 0.2^a^	8.0 ± 0.2^a^	9.0 ± 0.0*	1.7 ± 0.1^bc^	4.6 ± 0.3^ab^
*R. babjevae* 109	1.5 ± 0.1^f^	3.3 ± 0.3^d^	9.0 ± 0.0*	1.0 ± 0.2^e^	4.3 ± 0.3^b^
*P. rhodozyma* 110	3.1 ± 0.3^c^	5.5 ± 0.5^c^	9.0 ± 0.0*	1.3 ± 0.2^de^	5.0 ± 0.4^a^
*C. psychroaquaticum* 111	3.5 ± 0.3^bc^	8.0 ± 0.4^a^	9.0 ± 0.0*	1.8 ± 0.1^bc^	5.0 ± 0.1^a^
*C. psychroaquaticum* 112	5.0 ± 0.5^a^	7.0 ± 0.2^b^	9.0 ± 0.0*	1.5 ± 0.2^cd^	5.0 ± 0.3^a^
*C. capitatum* 113	2.5 ± 0.1^d^	7.5 ± 0.3^ab^	9.0 ± 0.0*	1.3 ± 0.1^de^	5.0 ± 0.2^a^
*P. rhodozyma* 114	2.7 ± 0.2^cd^	7.5 ± 0.2^ab^	9.0 ± 0.0*	1.5 ± 0.2^cd^	4.8 ± 0.3^ab^
*R. colostri* 115	2.5 ± 0.2^d^	8.0 ± 0.2^a^	9.0 ± 0.0*	1.3 ± 0.2^de^	5.0 ± 0.4^a^
*C. infirmominiatum* 116	5.0 ± 0.4^a^	8.0 ± 0.5^a^	9.0 ± 0.0*	1.3 ± 0.1^de^	5.3 ± 0.3^a^
*R. colostri* 117	5.0 ± 0.5^a^	7.0 ± 0.1^b^	9.0 ± 0.0*	1.4 ± 0.2^cd^	5.3 ± 0.4^a^
*R. colostri* 118	2.0 ± 0.1^e^	7.5 ± 0.4^ab^	9.0 ± 0.0*	1.4 ± 0.2^cd^	5.0 ± 0.2^a^
*C. capitatum* 119	5.0 ± 0.4^a^	8.0 ± 0.2^a^	9.0 ± 0.0*	1.7 ± 0.3^bc^	5.0 ± 0.4^a^
Control	5.0 ± 0.2^a^	8.1 ± 0.3^a^	9.0 ± 0.0*	3.2 ± 0.2^a^	5.3 ± 0.3^a^

Indexes a,b,c… means homogeneous groups determined by Tukey’s test (one-way analysis of variance)

*No significant differences

The yeast *R. mucilaginosa* was characterised by the highest antimicrobial activity against the mould *Botrytis cinerea*. Compared to the control culture (8.0 cm), the mould mycelium was the smallest (0.7–1.3 cm) on the plates after cultivation with these strains. Good antagonistic activity (1 cm) against *B. cinerea* was also shown by the isolate of *R. glutinis* (CMIFS 103), while much weaker activity was shown by *R. babjevae* CMIFS 109 (3.3 cm). A study by Zhang et al. (2014) also found the ability of *R. mucilaginosa* to reduce the growth of the mould *B. cinerea*, and additionally showed an increase in this activity when the yeast was grown in chitosan-containing media. Zhang et al. (2010) studied the antagonistic activity of yeast *R. glutinis* and salicylic acid against *B. cinerea*. More effective reduction of mould growth was achieved using both yeast and acid than with only yeast. Two strains of *P. rhodozyma* (CMIFS 102 and 110) inhibited the growth of *B. cinerea* (5.5 cm) with the same activity, while the third strain (CMIFS 114) did not limit its expansion (7.5 cm). The strains of the genus *Cystofilobasidium, Rhodosporidiobolus, Cystobasidium, Buckleyzyma* and *Symmetrospora* did not significantly limit the growth of *B. cinerea*.

None of the red yeast strains tested in this study showed antagonistic properties towards *Penicillium expansum*. However, in the work of Zara et al. (2020), during the cultivation of various red yeasts, one of the strains of *R. mucilaginosa* DiSVAC71t0a was found to be able to significantly reduce (> 50%) the development zones of *P. expansum* mould developing on apples. According to Qian et al. (2020), the mechanisms associated with the antagonistic activity of *R. mucilaginosa* include a faster growth rate on the fruit surface, thanks to which they compete for nutrients and space with *P. expansum*, as well as the production of specific enzymes (chitinase and β-1,3-glucanases). The yeast *R. mucilaginosa* also has the ability to degrade the toxin produced by this mould, i.e. patulin.

The tested yeast strains inhibited the growth of the mould *Alternaria solani* to varying degrees. The best antagonistic effect was shown by yeast belonging to the genus *Rhodotorula* because the mycelium of this mould after cultivation with yeast compared to the control (3.0 cm) was much smaller: from 0.6 (CMIFS 004 and 098) to 1.0 cm (CMIFS 109). A study by Yan et al. (2014) showed that rhamnolipids produced by *Pseudomonas aeruginosa* increased the antagonistic activity of *R. glutinis* against *Alternaria alternata* when tested on cherry tomatoes. All strains belonging to such genera as *Cystofilobasidium, Phaffia* and *Symmetrospora* were characterised by a similar ability to limit the growth of *A. solani*. The diameter of the mycelia on the media inoculated with this yeast ranged from 1.3 to 1.7 cm. *Rhodosporididiobolus* yeast, depending on the strain, showed a more diverse antagonistic activity (1.3–2.2 cm) against *A. solani*. In the case of *C. psychroaquaticum* strains, smaller growth zones of this mould were also observed, ranging from 1.5 to 2.0 cm. The *B. aurantiaca* strain CMIFS 105 also inhibited the growth (2.0 cm) of *A. solani*.

As with other moulds tested, the smallest mycelium of *Fusarium solani* (2.6–4 cm) was observed on plates after co-culture with the yeast *R. mucilaginosa*. The strains *R. glutinis* (CMIFS 103) and *R. babjevae* (CMIFS 109) also showed antagonistic activity against this mould because the diameter of the mycelia was smaller than the control (5.3 cm) and was 3.5 and 4.3 cm, respectively. Among the yeasts belonging to *P. rhodozyma*, the CMIFS 102 strain was characterised by the highest antagonistic activity (4.0 cm). Other red yeast strains did not significantly limit the growth of *Fusarium solani*. Some strains of red yeast have the ability to bind mycotoxins produced by moulds of the genus *Fusarium*. Studies by Srinual et al. (2022) showed that supplementation of broiler chicken feed with red yeast *Sporidiobolus pararoseus* KM281507 attenuated toxicity mainly induced by zearalenone and deoxynivalenol and could potentially be used as a novel feed additive in the broiler industry.

The huge antagonistic potential of yeast is still widely researched and described in the literature. To date, several species of yeast, such as *Candida oleophila, Aureobasidium pullulans, Metschnikowia fructicola, Cryptococcus albidus* and *Saccharomyces cerevisiae*, have been used in commercial preparations used in the biocontrol of plant pathogens (Ma et al. 2023).

## Summary

Different strains of carotenoid yeast can be isolated from the natural environment of deciduous trees, specifically silver birch (*Betula pendula*). All tested red yeast strains showed the ability to biosynthesise carotenoids. Most of the tested yeasts can be considered oleaginous, because, after cultivation in a medium with an initial C/N = 70. The initial screening on the plates allowed the selection of eleven strains with lipolytic activity, nine strains producing amylases and two strains degrading gelatin. The tested red yeast strains were not efficient producers of extracellular polysaccharides and did not produce extracellular biosurfactants. None of the tested red yeast strains showed antagonistic properties towards *P. expansum*. In the case of the remaining moulds tested (*A. niger, B. cinerea, A. solani* and *F. solani*), the yeast of the genus *Rhodotorula* had the best ability to limit their growth. In conclusion, the conducted research allowed the isolation and selection of new strains of red yeast with biotechnological potential for the production of various valuable metabolites and as agents for mould biocontrol. In the longer term, it is necessary to conduct further research aimed at optimising the cultivation conditions of selected strains in order to maximise the synthesis of metabolites and the use of waste substances as components of culture media, which would significantly reduce their production costs. For potentially antagonistic yeasts, it is necessary to perform in vivo tests on plant materials, where moulds cause high economic losses during post-harvest storage.

## Data Availability

Data will be made available on request.
